# Single-Cell Transcriptomic Analysis Reveals Distinct Pathogenic Subtypes in Hepatocyte- and Cholangiocyte-Directed Autoimmune Liver Injury

**DOI:** 10.3390/ijms27209323

**Published:** 2026-10-20

**Authors:** Peiyi Zhao, Li Zhao, Huayang Zhang, Xiting Pu, Jun Qian, Yudong Zhao, Zhengrui You, Xiong Ma

**Affiliations:** Division of Gastroenterology and Hepatology, Shanghai Institute of Digestive Disease, Renji Hospital, School of Medicine, Shanghai Jiao Tong University, 145 Middle Shandong Road, Shanghai 200001, China; paisleypep@sjtu.edu.cn (P.Z.); zhaoligf@163.com (L.Z.); zhy123456@sjtu.edu.cn (H.Z.); xitingpu@163.com (X.P.); qianj1098@gmail.com (J.Q.); dongkakaka@sjtu.edu.cn (Y.Z.)

**Keywords:** autoimmune liver disease, Kupffer, bone morphogenetic protein, endoglin

## Abstract

Autoimmune liver diseases (AILDs) involve a group of distinct disorders, but the differences in pathogenic subpopulations that drive hepatocyte or bile duct injury remain unclear. This study characterizes immune niche heterogeneity across AILD subtypes using single-cell RNA sequencing and verifies findings with animal models. We identified distinct immune cell states and functions by analyzing the heterogeneity of T cell and macrophage subsets and the impact of non-immune cell-derived signals on macrophage identity. This allowed us to characterize two distinct immunopathogenic ecotypes between hepatitis and cholestatic phenotypes. Mechanistic analysis predicted the Bmp–Acvrl1/Eng–Fosb signaling axis as a critical transcriptional regulator driving the repair phenotype of macrophages. Notably, Eng (endoglin) emerged as a regulatory receptor controlling macrophage phenotypic transition, especially during the recovery phase of cholestatic disease. Collectively, this study provides a comprehensive atlas of immune cell niches across AILDs, offering new mechanistic insights into disease heterogeneity and identifying macrophage maturation as a promising target for stratified therapeutic intervention in cholestatic disease.

## 1. Introduction

Autoimmune liver diseases (AILDs) comprise a group of chronic, progressive liver disorders that result from a collapse in immune tolerance. Key types include primary biliary cholangitis (PBC), primary sclerosing cholangitis (PSC), and autoimmune hepatitis (AIH), each distinguished by different cellular targets. PBC and PSC primarily affect biliary epithelial cells, leading to cholestatic injury, and AIH mainly targets hepatocytes. Both genetic susceptibility and environmental factors contribute to the disruption of self-tolerance, ultimately initiating abnormal immune responses in the liver [1].

These distinct injury patterns are significantly associated with heterogeneity in treatment responses and clinical outcomes. In standard clinical practice, AIH is treated with corticosteroid-based immunosuppression, and ursodeoxycholic acid is the first-line therapy for PBC, while effective treatments for PSC remain limited. Notably, diseases characterized by cholangiocyte injury are often refractory to conventional immunosuppressive therapy and prone to progression to biliary fibrosis and cirrhosis [2]. It remains unclear whether these distinct pathological phenotypes and treatment responses arise from different pathogenic subpopulations in the intrahepatic immune microenvironment.

Evidence indicates that AILDs involve a complex immune network in which innate and adaptive immunity act in concert. Among innate immune populations, hepatic macrophages exhibit substantial plasticity. In different AILDs models, these cells exhibit distinct functional roles, capable of exerting either pathogenic effects or contributing to tissue repair processes. In the acute hepatitis model, macrophage subsets contribute to the clearance of necrotic hepatocytes and subsequent tissue repair [3]. In cholestatic injury, macrophages activate chemotactic and migratory programs to sense tissue damage and recruit additional immune cells [4]. A recent study has identified an adaptive immunosuppressive signature in lipid-associated macrophages (LAMs) [5], whereas others suggest that these cells exert pro-inflammatory effects, driving ductular reactions and periportal inflammation [6]. Analysis of AILD patients demonstrates that PSC exhibits substantial macrophage heterogeneity, particularly in monocyte-like pro-fibrotic populations [7]; the macrophages of PBC patients show decreased scavenger and increased inflammatory functions [8]. Since there is no unified atlas for defining macrophage subsets in AILDs, it is difficult to systematically compare hepatic macrophages in hepatocellular injury versus cholestatic injury to determine whether conserved disease-specific subsets exist, or if macrophages in these two conditions exhibit different subset compositions and functions.

Adaptive immune cells, particularly CD$8^{+}$ T cells, can also mediate hepatocyte and bile duct injury. In mouse models, depletion of CD$8^{+}$ T cell populations or inhibition of their activation both result in significant histological improvement in hepatic tissue [9,10]. Given that glucocorticoid therapy can significantly induce T cell apoptosis [11], yet AIH patients and those with PBC/PSC still exhibit heterogeneous responses to immunosuppressive therapy, whether this variability stems from differential T cell involvement within the immunopathogenic network remains unclear.

Although high-throughput studies of individual AILDs are increasing, existing evidence remains limited to single disease entities or injury models. Direct comparisons between hepatocyte-centered and cholangiocyte-centered AILDs are insufficient, limiting our insight into their distinct immune landscapes and the mechanisms underlying heterogeneous treatment responses. To address this gap, we generated scRNA-seq data from the AAV8-IL12 mouse hepatitis model and integrated it with public datasets, yielding a cross-model intrahepatic cells atlas. We first confirmed that the AAV8-IL12 model recapitulates the T cell–dominant landscape reported in human AIH, characterized by CD$8^{+}$ T cells functioning as pathogenic subsets. Concurrently, engrafted monocytes were able to replenish the KC pool, acquiring a mature phenotype with reparative features in AIH models. Conversely, the macrophages dominating cholestatic liver injury remain arrested in an immature, pro-inflammatory monocyte-like state, and the same polarity was validated in human AILD samples. We predict that the Bmp-Acvrl1/Eng-Fosb module promotes macrophage maturation. Impaired BMP signaling sensitivity in cholestasis hinders macrophage differentiation toward a mature KC-like phenotype; these macrophages in cholestasis exhibit lower Eng expression, which can recover during the repair phase. Taken together, macrophage sensitivity to BMP signaling correlates with homeostatic phenotype, suggesting that targeting macrophage programming may provide a more effective treatment for cholestatic disease than broad immunosuppression.

## 2. Results

### 2.1. Cross-Model scRNA-Seq of Liver CD${\mathrm{45}}^{+}$ Cells Reveals Heterogeneous Immune Subsets

**Figure 1:**
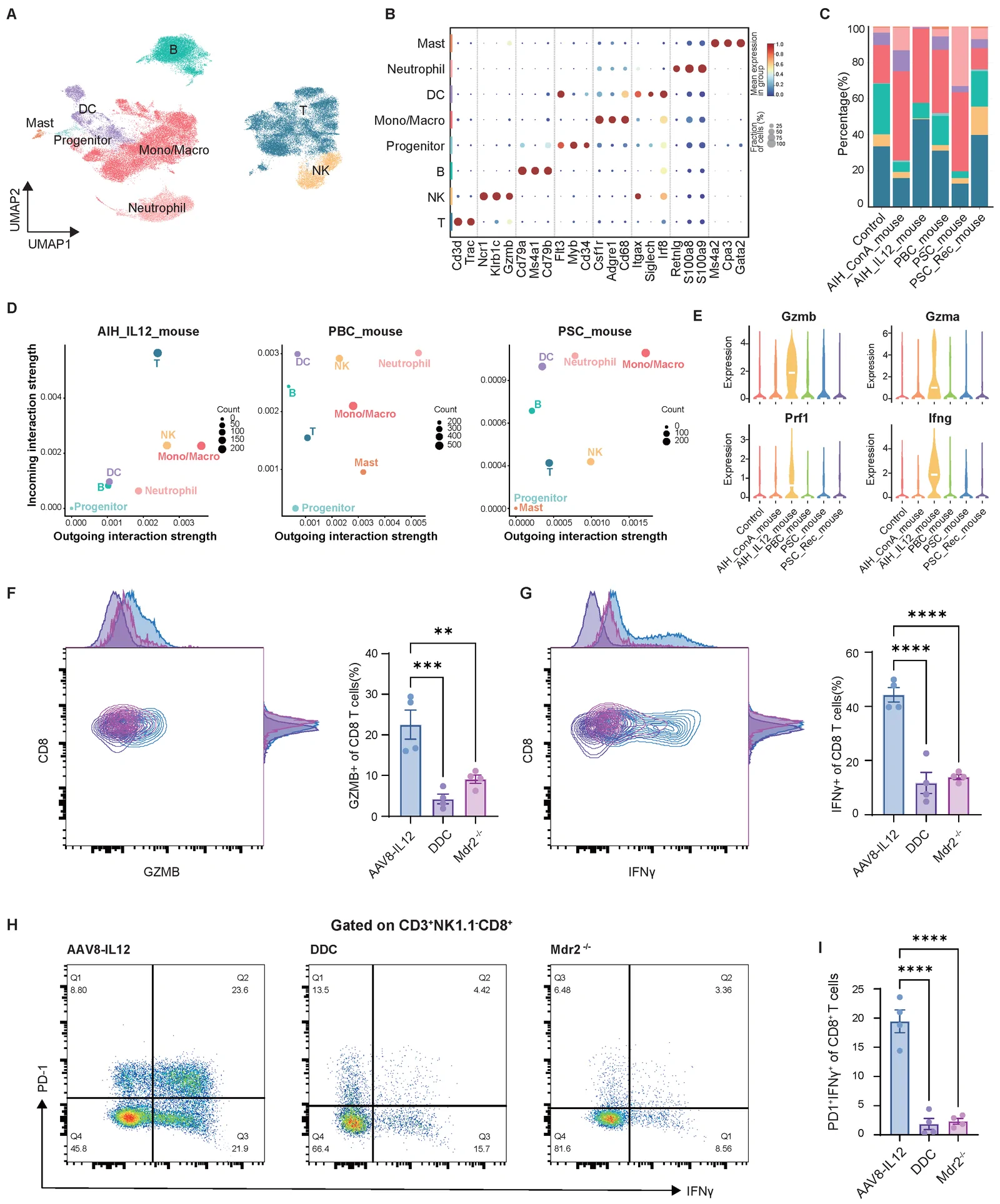
The single-cell atlas of CD$45^{+}$ cells. (**A**) UMAP plot of immune cell clusters. (**B**) Bubble plot of immune cell type markers. (**C**) Bar plots showing the cell proportions of liver immune cell subsets. (**D**) Scatter plots showing the overall outgoing (x-axis) and incoming (y-axis) communication probability for each cell type; dot size is proportional to the number of inferred ligand-receptor links associated with each cell type. (**E**) Violin plot showing effector gene expression of T cells. Representative flow cytometry plots of (**F**) CD$8^{+}$${\mathrm{GZMB}}^{+}$ T cells. (**G**) ${CD8}^{+}$IFN$\gamma^{+}$ T cells. (**H**,**I**) PD-$1^{+}$IFN$\gamma^{+}$ T cells among ${CD8}^{+}$ T cells. Statistical significance was determined by one-way ANOVA with Tukey’s multiple comparisons test. Data are presented as Mean ± SEM, *n* = 4 per group. Significance levels were set at ** p<0.01, *** p<0.001, and **** p<0.0001.

To better understand the heterogeneity of intrahepatic infiltrating immune cell subsets across autoimmune liver diseases, we integrated liver CD$45^{+}$ scRNA-seq datasets from multiple murine models, including public repositories and our own AAV8–IL12 model. After batch correction and integration, the combined CD$45^{+}$ compartment was analyzed by unsupervised dimensionality reduction and clustering (Figure 1A and Figure S1A). Based on key lineage markers, eight major immune cell lineages were identified (Figure 1B). The analysis of subpopulation proportions showed that, in the IL12-mediated hepatitis model, T cells constituted a significantly larger fraction than in cholestasis models. The Mono/Macro group was the predominant population in both hepatitis and cholestasis mice (Figure 1C).

CellChat was used to identify the roles of immune cell subpopulations in the cell–cell communication network, revealing different functions for T cells across AILD models. In the AAV8-IL12 model, T cells exhibited a high incoming strength, highlighting their role as key downstream effectors in hepatitis, while in cholestasis mice, T cells appeared more as bystanders (Figure 1D). Myeloid cells (Mono/Macro or Neutrophil) showed significant outgoing strength in all three disease models, indicating they are primary upstream drivers of inflammation. These findings suggest distinct pathogenic mechanisms: the chronic AIH model is mainly driven by T cell-mediated adaptive immunity, whereas the cholestasis model is dominated by myeloid cell-driven innate-like immunity. More specifically, in the AAV8-IL12 model, effector memory (EM) and Cytotoxic/Cycling CD8 T cells expanded greatly, implicating CD8-mediated cytotoxicity in hepatitis (Supplementary Figure S2A–C). These T cells also showed higher cytotoxic/pro-inflammatory marker expression than in other models (Figure 1E). To validate the scRNA-seq results, we used flow cytometry to compare effector molecule expression in liver-infiltrating CD$8^{+}$ T cells across the AAV8-IL12 hepatitis model and two cholestasis models (DDC diet and Mdr$2^{-/-}$). CD$8^{+}$ T cells from hepatitis mice showed higher GZMB (Figure 1F) and IFNγ (Figure 1G) levels than those from cholestatic mice, confirming their enhanced cytotoxic and pro-inflammatory phenotype. Notably, a unique subset of CD$8^{+}$ T cells was identified in AAV8-IL12 mice that resembles the CD8 EM profile with high expression of PD-1. Indeed, these cells are not fully exhausted but still retain functional capacity, including IFNγ production, indicating that PD-1 expression here may signify activation or regulation rather than terminal exhaustion (Figure 1H,I).

Cell–cell interaction analysis also demonstrated that macrophages drive both the inflammatory response and negative feedback regulation to impress T cells. Notably, there were relatively prominent protection mechanisms in AIH models with high expression of Pdcd1 and Adora2a (Supplementary Figure S2D,E). Macrophages limit T cell overactivation through the Cd274/Pdcd1 checkpoint, and the DAMP-activated EntPD-1/Adora2a pathway induces metabolic reprogramming to suppress T cell energy use and impair their effector functions [12].

### 2.2. Macrophages Infiltrating the Liver of AILDs Mice Have Varying Differentiation States

**Figure 2:**
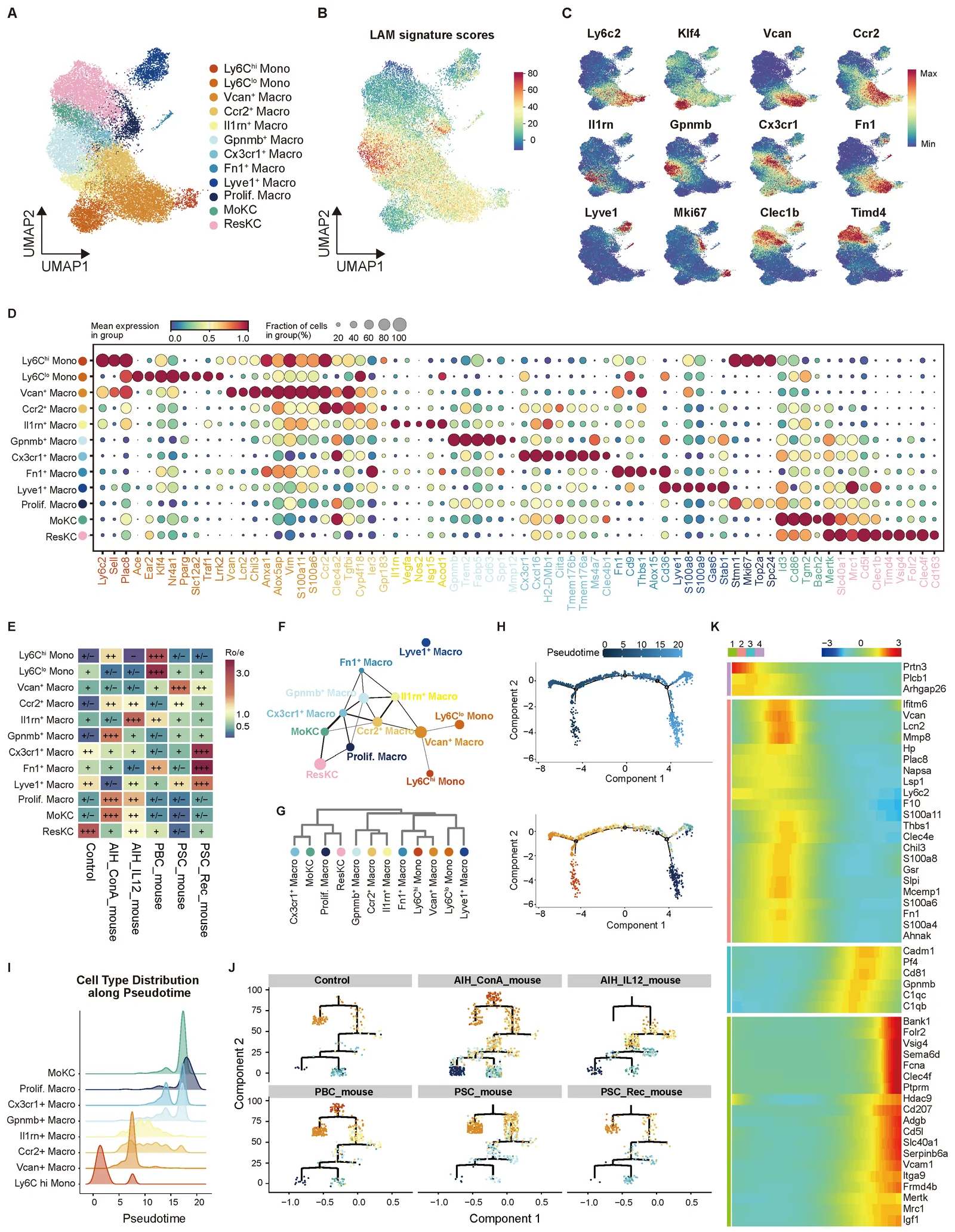
Macrophage heterogeneity across different AILDs models. (**A**) UMAP plot of Mono/Macro re-clusters. (**B**) UMAP plot showing LAM gene signature. (**C**) UMAP plots showing the marker gene of each subtype. (**D**) Bubble plot showing markers of Mono/Macro subclusters. (**E**) Group preference estimated by Ro/e score. +++, Ro/e >2; ++, 1 < Ro/e ≤2; +, 0.5 < Ro/e≤ 1; +/−, 0 < Ro/e ≤ 0.5; −, Ro/e = 0. (**F**) PAGA analysis shows the topological connectivity among subtypes. (**G**) Hierarchical analysis. (**H**) Developmental trajectory of Ly6$C^{hi}$ Mono, MoMF, MoKC in livers. (**I**) Ridge plot showing Mono/Macro subtype distribution along pseudotime. (**J**) Tree structure of the trajectory. (**K**) Heatmap showing the top 50 significant genes, hierarchically clustered based on pseudotime-dependent expression (FDR <0.01).

To further delineate the upstream drivers of myeloid cell states, we performed high-resolution subclustering of Mono/Macro populations, identifying 12 distinct transcriptional groups. These could be categorized into three main classes: 2 monocyte subsets, 8 monocyte-derived macrophage (MoMF) subsets, and 2 Kupffer cell (KC) subsets (Figure 2A). Classical Ly6$C^{hi}$ monocytes showed inflammatory traits and high proliferation, while non-classical Ly6$C^{lo}$ monocytes displayed patrolling behavior. The MoMFs included eight states. Among them, ${\mathrm{Gpnmb}}^{+}$ macrophages were enriched for LAM features (Figure 2B). Fn$1^{+}$ macrophages shared characteristics with peritoneal macrophages, showing high expression of Gata6 and Alox15 [13] (Supplementary Figure S3A). Lyve$1^{+}$ macrophages expressing Cd206 and Esam remain controversial as embryonic KC2 [14,15] or marker cross-contamination of doublets [13] (Supplementary Figure S3B). Two KC-related populations were identified: a Timd$4^{-}$ monocyte-derived Kupffer-like cells (MoKCs) expressing Clec1b, and a resident Timd$4^{+}$ Kupffer cell (ResKC) subset expressing Timd4, Vsig4, Folr2, Clec4f, and Cd163 (Figure 2C,D). This comprehensive single-cell framework provides a standard reference for comparing macrophage states across autoimmune liver disease models and between inflammatory and recovery phases.

Across all disease models, KC numbers declined consistently, yet the compensatory subclusters for this loss diverged dramatically depending on disease type (Figure 2E). In the cholestasis models, the intrahepatic macrophage subpopulations were dominated primarily by Ly6$C^{hi}$ monocytes and transitional ${\mathrm{Vcan}}^{+}$ macrophages. The PSC recovery phase (DDC withdrawal) revealed Cx3cr1$1^{+}$ macrophages specifically expanded to actively replenish the KC pool. Conversely, in both AIH models, this loss was accompanied by marked expansion of MoKC and proliferating macrophages, indicating that recruited monocytes were actively acquiring a KC-like identity and undergoing local proliferation to replenish the niche. During ConA-induced acute hepatitis recovery, ${\mathrm{Gpnmb}}^{+}$ macrophages with LAM features also increased significantly. Both Cx3cr$1^{+}$ Macro and MoKC express Cd207 (Supplementary Figure S3C), resembling liver capsular macrophages (LCMs) [16]. Based on PAGA analysis (Figure 2F) and hierarchical clustering (Figure 2G), which reflect developmental relationships among the 12 clusters, these two populations were closely linked. This suggests they may share similar functions.

To explore the differentiation state of engrafted inflammatory Ly6$C^{hi}$ monocytes in the liver, trajectory analysis was conducted on the remaining eight subpopulations (excluding Ly6$C^{lo}$ Mono, Timd$4^{+}$ KC, Lyve$1^{+}$ Macro, and Fn$1^{+}$ Macro). Pseudotime analysis identified a continuous differentiation trajectory. Cholestatic mice exhibited a higher proportion of early differentiated macrophages, whereas hepatitic mice displayed a predominance of later-stage macrophages (Figure 2H–J). Along this trajectory, four sequential gene expression modules were defined (Figure 2K). The initial stage was characterized by expression of early myeloid progenitor genes (Prtn3, Arhgap26). Subsequently, cells transitioned into an acute inflammatory monocyte state, marked by upregulation of Ly6c2, acute-phase proteins (Lcn2, Hp), and S100 alarmins (S100a8/a11). This inflammatory program was subsequently silenced and replaced by an immune resolution phenotype (Pf4, C1qb, C1qc, Gpnmb), indicating a shift toward complement-mediated efferocytosis and clearance of apoptotic debris, thereby functionally bridging inflammation and repair. In the terminal state, cells acquired a mature tissue-resident macrophage identity, co-expressing canonical residency markers (Mrc1, Vsig4, Folr2, Clec4f), efferocytosis receptors (Mertk), tissue-repair mediators (Igf1), and genes involved in adhesion and metabolic adaptation (Vcam1, Itga9, Slc40a1), consistent with a homeostatic phenotype. Collectively, this trajectory illustrates a progressive phenotypic shift from early inflammatory readiness to late-stage tissue maintenance, demonstrating the reprogramming of monocytes into niche-adapted resident macrophages. Notably, this reprogramming process is abnormal in cholestatic mice compared to hepatitis mice.

### 2.3. Distinct Macrophage Phenotypes Between Hepatitis and Cholestasis Models In Vivo

**Figure 3:**
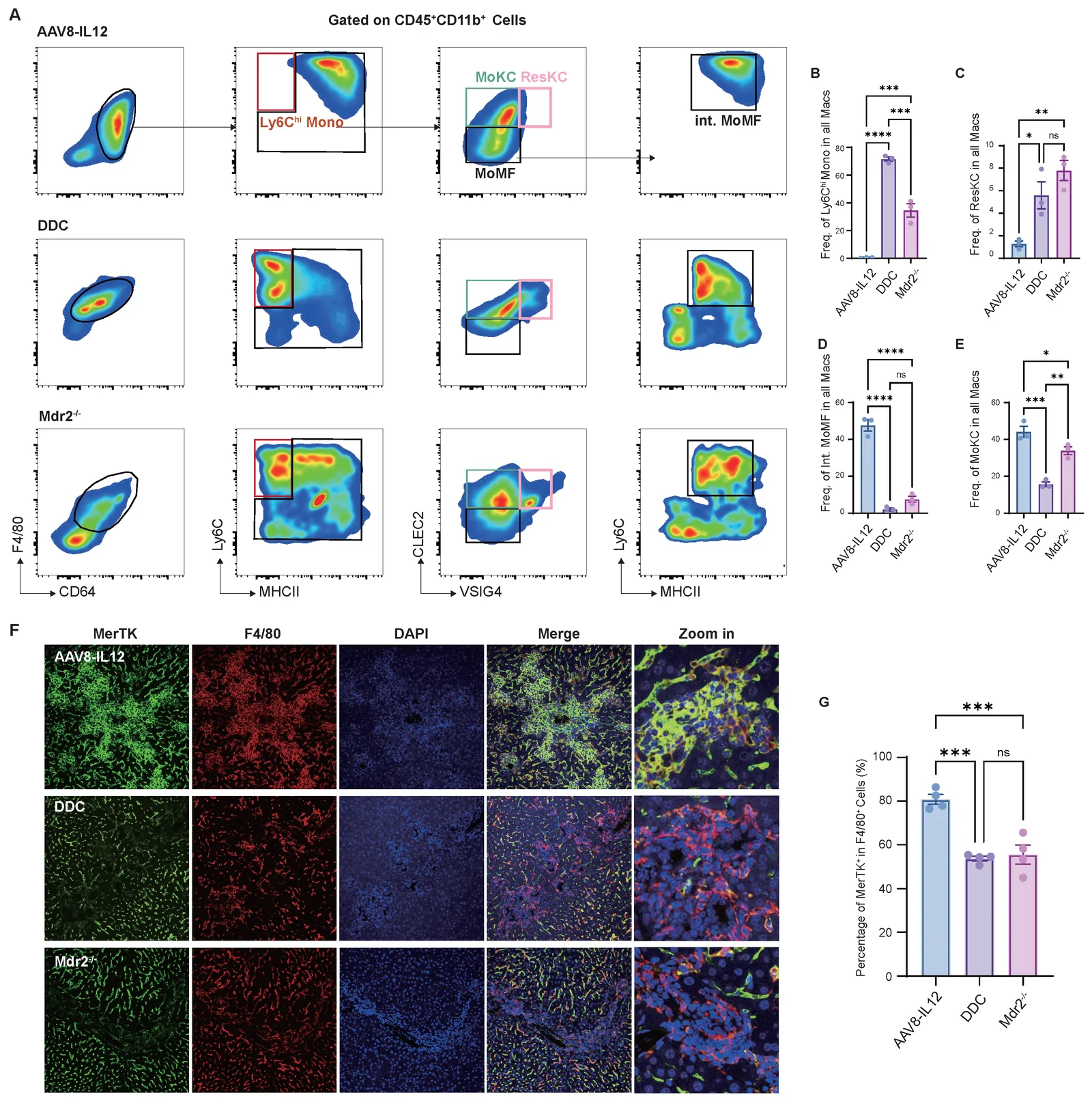
In vivo validation of hepatic macrophage phenotypes. (**A**–**E**) Representative flow cytometry plots showing the frequency of macrophage subtypes. (**F**,**G**) Representative immunofluorescence images and quantification of the proportion of F4/$80^{+}$${\mathrm{MerTK}}^{+}$ macrophages. Statistical significance was determined by one-way ANOVA with Tukey’s multiple comparisons test. Data are presented as Mean ± SEM, *n* = 4 per group. Significance levels were set at * p<0.05, ** p<0.01, *** p<0.001, and **** p<0.0001.

To verify the differences in macrophage maturity between hepatitis and cholestatic mice, intrahepatic mononuclear cells were isolated for flow cytometry to compare the composition of the CD6$4^{+}$F4/$80^{+}$ mono/macrophages population. Monocyte-like cells (Ly6$C^{hi}$${\mathrm{MHCII}}^{lo}$) were significantly higher in cholestatic mice (DDC diet and Mdr$2^{-/-}$) than in the IL12 model (Figure 3A,B). ResKC (VSIG$4^{hi}$) was markedly reduced in AAV8-IL12 mice (Figure 3C), accompanied by a large number of transitional macrophages (CLEC$2^{lo}$Ly6$C^{hi}$${\mathrm{MHCII}}^{hi}$) and replenishment of CLEC$2^{hi}$MoKC (Figure 3D,E). Furthermore, tissue immunofluorescence staining demonstrated a reduced proportion of MerTK-positive cells among F4/$80^{+}$ macrophages in cholestatic mice, indicating impaired MerTK expression in macrophage populations (Figure 3G). Multiple studies have shown that antibody-mediated blockade or knockdown of the MerTK receptor in mouse primary macrophages or cell lines impairs efferocytosis [17,18]. In line with this, our observation of lower MerTK expression in cholestatic mice suggests a potential association with impaired efferocytosis. In the AAV8-IL12 model, hematoxylin and eosin (HE) staining and immunofluorescence of liver tissue revealed crown-like structures around portal tracts and granuloma-like lesions between lobules; both were aggregation foci of F4/$80^{hi}$${\mathrm{MerTK}}^{hi}$ cells (Supplementary Figure S4A,B). Although crown-like structures typically surround necrotic areas and are involved in dead cell clearance, as described in ConA-induced acute liver injury [3], the interlobular foci observed in AAV8-IL12 exhibit more distinctive features.

### 2.4. Macrophages in Early and Late Stages of Differentiation Exhibit Distinct Pro-Inflammatory and Homeostasis-Maintaining Profiles

**Figure 4:**
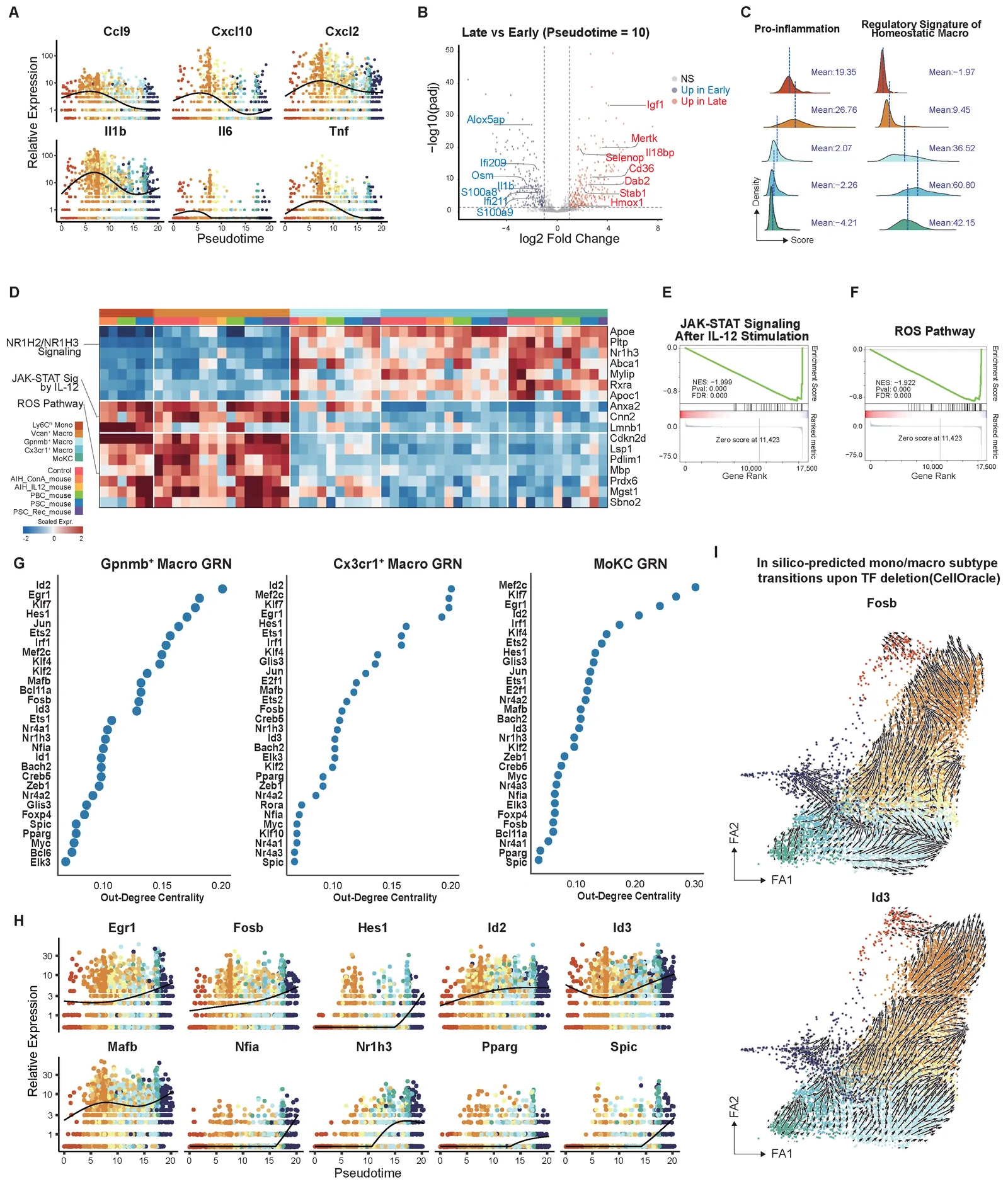
Function and regulatory mechanism analysis. (**A**) Pro-inflammatory factor and chemokine expression trends over pseudotime. (**B**) Volcano plot of DEGs between early/late stages. Paired early/late pseudo-bulks per sample. SigDEGs:$\mathrm{FDR}<0.05,\;|\log_{2}\mathrm{FC}|>0.6,\;\mathrm{pct}.\;\mathrm{expressed}>30\%$. (**C**) Ridge plot showing Mono/Macro subtype distribution along gene signature scores. (**D**) Matrix plot illustrating leading genes of representative enriched pathways of pseudo-bulk per sample within cell type, excluding low-quality bulks. (**E**,**F**) Enrichment plots of MoKC. (**G**) Top 30 TFs ranked by out-degree centrality. (**H**) Up-regulated expression trends of TFs. (**I**) CellOracle in silico perturbation of Fosb/Id3; vectors show predicted cell-state shift direction.

To investigate how functional effector molecules vary across macrophage differentiation stages, we first examined how the expression levels of a panel of inflammatory chemokines changed along the pseudotime trajectory. We found that classic pro-inflammatory and chemotactic factors reached their peaks during early differentiation and then remained at low levels in later stages (Figure 4A). To enhance statistical power, we categorized the sample cells into early and late stages using a pseudotime threshold of 10 and performed differential analysis on pseudo-bulk data. This analysis demonstrated that late-stage cells exhibit strong expression of efferocytosis-related molecules (Mertk, Cd36, Stab1), antioxidants (Selenop, Hmox1), pro-repair factors (Igf1), and anti-inflammatory factors (Dab2, Il18bp). In contrast, early-stage cells display elevated levels of pro-inflammatory effectors (Il1b, Osm, S100a8, S100a9, Alox5ap) and interferon-responsive genes (Ifi209, Ifi211) (Figure 4B).

Given that Ly6$C^{hi}$ monocytes, ${\mathrm{Vcan}}^{+}$ macrophages, ${\mathrm{Gpnmb}}^{+}$ macrophages, Cx3cr$1^{+}$ macrophages, and MoKC populations exhibit distinct enrichment patterns across different mouse models and were distributed at opposite ends of the trajectory analysis branches, we subsequently focused our investigation of molecular functions on these cell types. To characterize the functional states of the five macrophage subclusters, we performed gene set scoring with previously established pro-inflammatory [19] and homeostatic macrophage gene signatures [20]. Ly6$C^{hi}$ monocytes and ${\mathrm{Vcan}}^{+}$ macrophages exhibited higher pro-inflammatory scores, while ${\mathrm{Gpnmb}}^{+}$, Cx3cr$1^{+}$ macrophages, and MoKC showed markedly elevated homeostatic macrophage scores (Figure 4C). We then performed Gene Set Enrichment Analysis (GSEA) based on pseudo-bulk and found that ${\mathrm{Gpnmb}}^{+}$, Cx3cr$1^{+}$ macrophages, and MoKC showed up-regulation of the LXR signaling pathway, yet Ly6$C^{hi}$ monocytes and ${\mathrm{Vcan}}^{+}$ macrophages exhibited marked reactive oxygen species (ROS) pathways and JAK-STAT signaling (Figure 4D–F). Taken together, the results indicated that early- and late-stage macrophages exhibit distinct functional states. Early-stage macrophages display pro-inflammatory properties and contribute to oxidative damage. Conversely, late-stage macrophages undergo metabolic and inflammatory alterations, transitioning from a pro-inflammatory state to a homeostatic phenotype. The early-stage macrophage subgroup is more prevalent in cholestatic liver injury, whereas the late-stage subgroup is more frequently observed in AAV8-IL12-induced hepatitis injury. These observations suggest that macrophages in cholestatic mice are more closely associated with immune imbalance.

To elucidate the transcription factors (TFs) driving phenotypic heterogeneity among macrophage subpopulations, we applied CellOracle to reconstruct gene regulatory networks (GRNs) in three late-stage macrophage clusters. The top 30 genes ranked by out-degree centrality identified several TFs with well-documented roles in macrophage biology, but their context-dependent roles remain unknown in AILDs (Figure 4G). We further narrowed down the candidates by selecting TFs linked to favorable phenotypes and whose expression progressively rose along pseudotime toward late-stage maturation (Figure 4H). Based on their established functions, these TFs broadly fall into three groups. Egr1, Hes1, Pparg, Spic, and Fosb are associated with alternative polarization (M2) and anti-inflammatory programs [21,22,23,24,25]. Id2 and Nfia are associated with terminal differentiation, though their macrophage-specific roles remain less defined [26,27]. As Id3, Mafb, and Nr1h3 have established roles in tissue-resident macrophage identity [28,29,30], the shared expression pattern alongside the trajectory of the remaining seven TFs raises the possibility that they may also contribute to the acquisition of the KC-like profile from engrafting monocytes. To evaluate the potential impact of these candidate TF deficiencies on KC identity, we performed perturbation modeling using CellOracle and visualized the results on a force-directed graph (FA1/FA2) initialized by PAGA. This analysis correctly recapitulated the known role of Id3, used as a positive control, in maintaining tissue-resident Kupffer identity. Among the remaining TF candidates, only Fosb depletion predicted comparable transcriptional changes from mature Kupffer-like toward naive monocyte-like phenotypes (Figure 4I). We speculated that Fosb, alongside Id3, may regulate transcriptional programs critical for maintaining KC identity.

### 2.5. Impaired Macrophage BMP Signaling Sensitivity in Cholestasis Models

**Figure 5:**
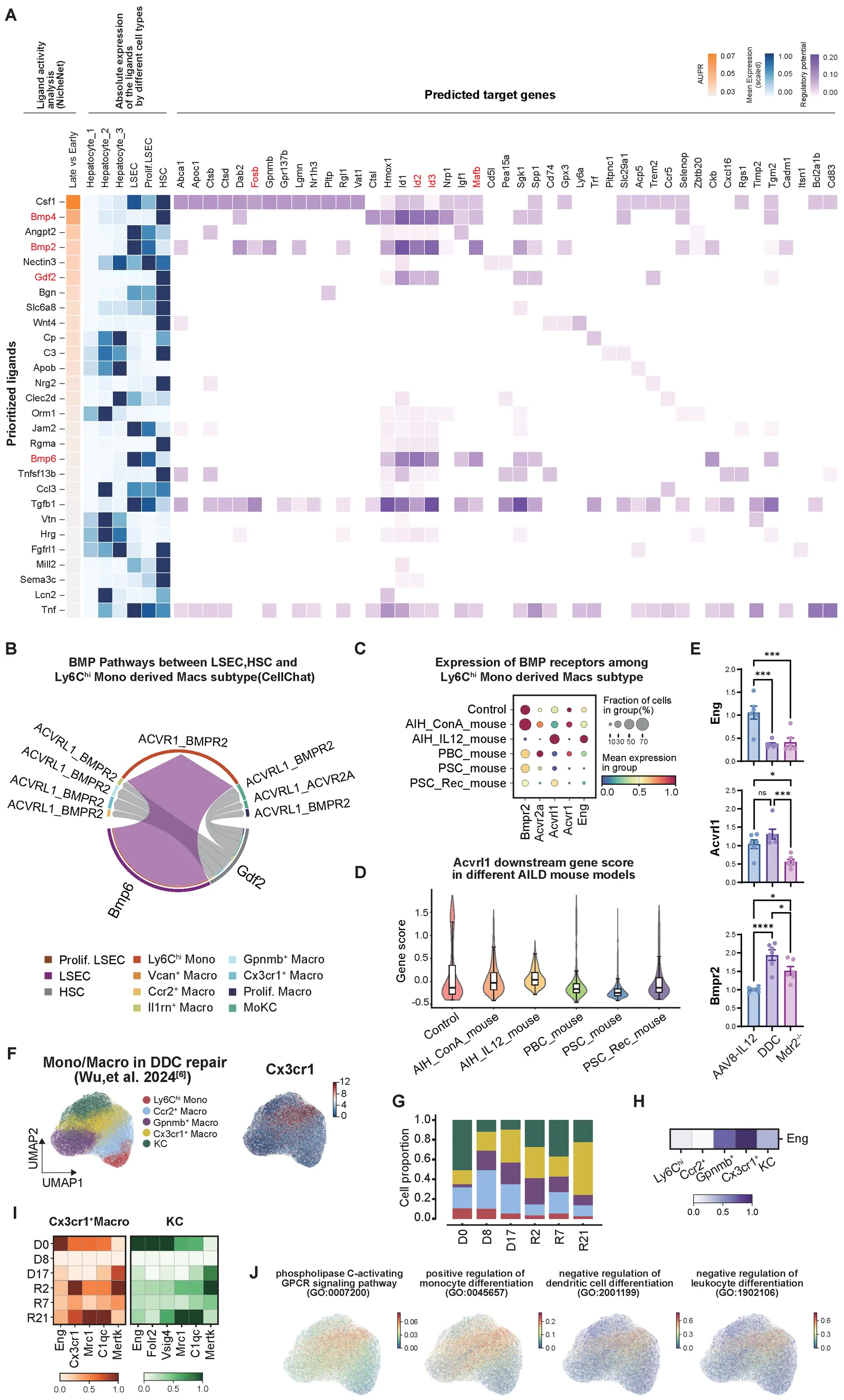
Regulation of macrophages by non-immune cells. (**A**) Matrix plot showing the NicheNet analysis of the upstream ligand from hepatocytes, LSEC, HSC, and predicted target genes of macrophages. (**B**) Chord diagram showing the BMP-BMPR interaction pairs. (**C**) Bubble plots showing BMPR gene relative expression. (**D**) Violin plot showing Acvrl1 downstream gene scores among different groups. (**E**) RT-qPCR showing mRNA expression levels of endoglin, Acvrl1, and Bmpr2. β-actin was used as a control. Data are presented as Mean ± SEM, *n* = 6 per group. Significance levels were set at * p<0.05, *** p<0.001, and **** p<0.0001. (**F**) UMAP plot showing the independent validation set of macrophage subsets [6]. (**G**) Bar plots showing the cell proportion of macrophage subsets at different times; D means the damage phase, and R means the recovery phase. (**H**) Matrix plot showing the relative expression of endoglin in different subtypes. (**I**) Matrix plots showing the relative expression of genes in Cx3cr$1^{+}$ Macro and KC. (**J**) UMAP plots showing Cx3cr$1^{+}$ macrophage-enriched pathway.

Previous studies have shown that liver non-immune cells contribute to the acquisition of KC identity by engrafted monocytes [13,29,31,32]. To find potential signals involved in the acquisition of KC-like identity, we integrated and clustered publicly available liver non-immune cell datasets from AILDs (Supplementary Figure S5A,B). We next performed NicheNet analysis to infer candidate ligand–receptor interactions as well as their downstream target genes that may regulate macrophage differentiation. DEGs between early- and late-stage macrophages, as defined by pseudotime analysis (Figure 4B), were used as the gene set of interest. We then assessed potential regulatory inputs from hepatocytes, liver sinusoidal endothelial cells (LSECs), and hepatic stellate cells (HSCs) to macrophages. This analysis highlighted several ligands associated with the bone morphogenetic protein (BMP) signaling pathway. In mice with AILDs, Bmp2 and Bmp6 were predominantly expressed by LSECs, and Bmp4 and Gdf2 (encoding Bmp9) were mainly derived from HSCs. This evidence suggests that BMP signaling may represent an important upstream regulatory pathway shaping macrophage identity in murine AILDs models (Figure 5A). To corroborate these predictions, we conducted CellChat analysis, which identified multiple BMP–BMPR-mediated interactions between HSCs/LSECs and MoMFs. Among these interactions, the ACVRL1–BMPR2 axis was particularly prominent in AILDs models (Figure 5B). Notably, several macrophage target genes predicted by NicheNet overlapped with transcription factors previously identified by CellOracle, including Fosb, Id2, Id3, and Mafb. This overlap supports a central role for these transcription factors in BMP-associated macrophage regulation in AILDs models. We further examined the expression of major BMP receptors and the co-receptor Eng (encoding endoglin) in macrophages across the different AILDs models. The results demonstrated that in cholestatic mice, the expression of the type II receptor (Bmpr2) for BMP signaling in macrophages remained normal. However, the type I receptor (Acvrl1) and its co-receptor (Eng), which are important for signal transduction and ligand capture [33], were found at lower levels (Figure 5C), suggesting a decrease in BMP signaling sensitivity. Liver macrophages from AAV8-IL12 models displayed an opposing receptor expression profile, characterized by elevated expression of Acvrl1 and Eng. To evaluate ALK1 (encoded by Acvrl1) downstream activity in macrophages, we used DEGs derived from macrophages of Alk$1^{fl/fl}$Clec4$f^{Cre}$ mice as the gene signature [34]. The ALK1 signaling score was lower in the cholestatic models than in the hepatitis model (Figure 5D). We then validated the in vivo expression of key BMP receptors in macrophages by RT-qPCR. The Eng expression in macrophage subsets of two cholestasis mouse models was lower than that in the AAV8-IL12 mouse model, and the expression level of Bmpr2 showed no significant abnormality, consistent with the scRNA-seq results (Figure 5E).

In addition, both Acvrl1 expression and the ALK1 downstream signaling score increased during the recovery phase of DDC models. To gain deeper insights into the dynamic patterns underlying this phenomenon, we validated our findings using an independent external DDC dataset [6] and extracted macrophage subsets solely (Figure 5F). Similarly, KC was observed to decrease compared to baseline after DDC intervention, and during the early repair phase, Cx3cr$1^{+}$ macrophages replenished the KC pool (Figure 5G). Taking into account the cellular expression profile of Eng, mature cell subsets are higher than monocyte-like subsets, with the highest expression in Cx3cr$1^{+}$ macrophages (Figure 5H). Longitudinal tracking in the DDC model showed that Eng expression decreased significantly in both Cx3cr$1^{+}$ macrophages and KCs during the injury phase compared to baseline. During the repair phase, Eng expression showed signs of recovery, coinciding with increased Cx3cr$1^{+}$ macrophage proportions and the upregulation of phagocytosis and tissue repair genes, and KCs adopted a reparative phenotype (Figure 5I). Following enrichment analysis of distinct cell subpopulations, results demonstrated that Cx3cr$1^{+}$ macrophages exhibited significant enrichment signatures across multiple critical signaling pathways. As the Cx3cr1 receptor itself is a G protein-coupled receptor (GPCR), this cell subpopulation also showed substantial enrichment in CGRP signaling; furthermore, the molecular programs associated with Cx3cr$1^{+}$ macrophages not only relate to monocyte differentiation promotion, but also exhibit negative regulatory trends on dendritic cell generation and lymphocyte differentiation (Figure 5J). These findings suggest that Cx3cr$1^{+}$ macrophage subpopulations may play an important role in regulating immune microenvironment signaling. Integrating evidence from both datasets, the dynamic trajectory of Eng expression, characterized by decline during injury and recovery during repair, was temporally synchronized with the expansion of Cx3cr$1^{+}$ Macrophages and the acquisition of a repair phenotype and immune-modulating effects.

**Figure 6:**
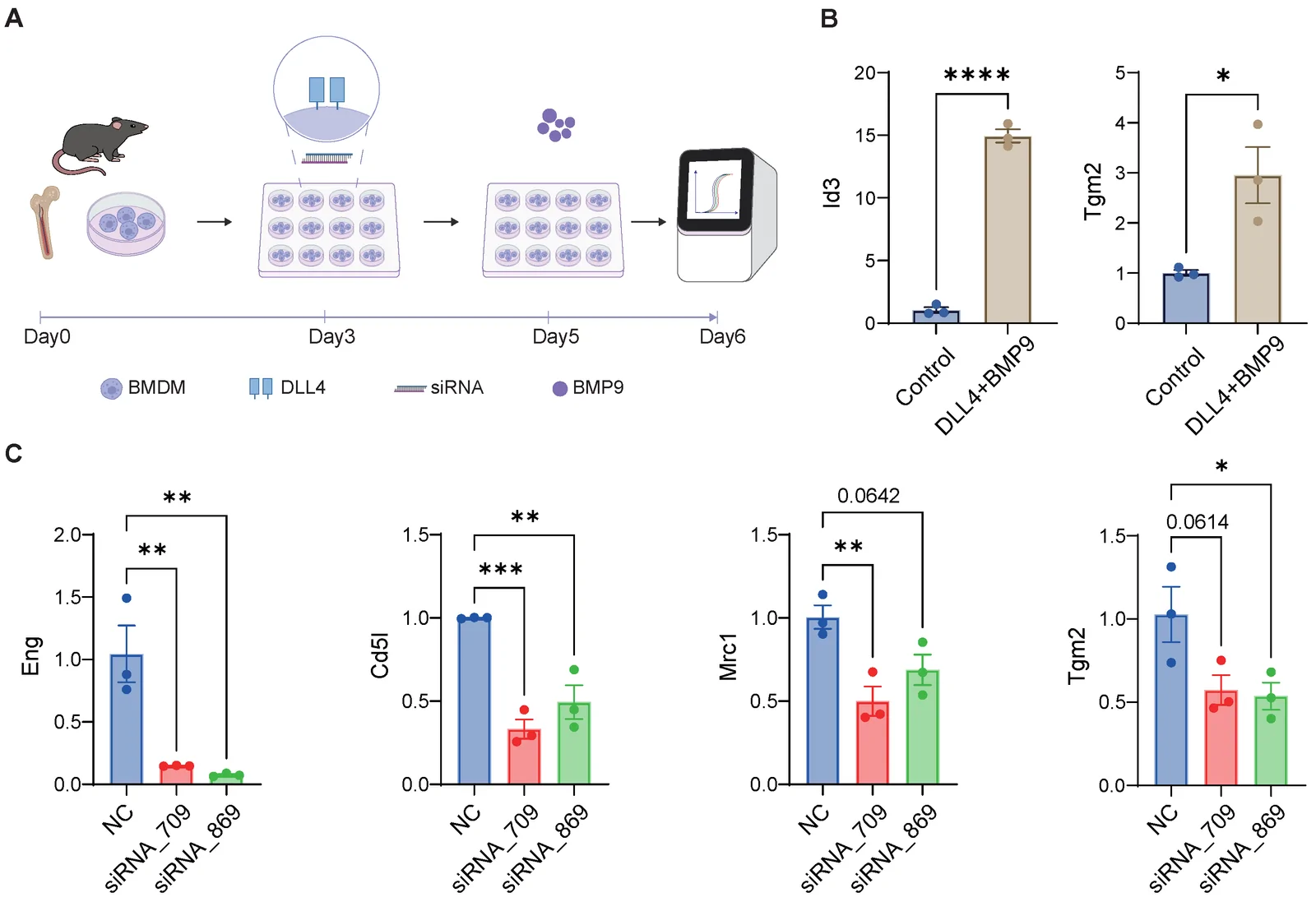
SiRNA against endoglin inhibits bone marrow progenitor cells from acquiring a KC-like phenotype. (**A**) Schematic diagram of the in vitro culture of bone marrow cells and the siRNA transfection process. RT-qPCR showing mRNA expression levels of (**B**) Id3 and Tgm2, the downstream genes of BMP9 signal. (**C**) Kupffer cell-related genes. β-actin was used as a control. Data are presented as Mean ± SEM, *n* = 3 per group. Significance levels were set at * p<0.05, ** p<0.01, *** p<0.001, and **** p<0.0001.

To examine endoglin’s role in macrophage maturation, and based on prior literature reporting that the acquisition and maintenance of Kupffer identity require the ligand DLL4 provided by LSECs to activate Notch signaling, as well as BMP9 signaling derived from HSCs, we employed an in vitro co-stimulation protocol with DLL4 plus BMP9 to mimic the differentiation process through which monocytes acquire a KC-like identity (Figure 6A). We then knocked down the Eng gene using siRNA to assess its effects on BMP9 sensitivity. The results showed that, compared with the conventional BMDM induction protocol, DLL4 and BMP9 co-stimulation significantly induced Id3, the key transcription factor determining the KC lineage, and also upregulated Tgm2, a downstream target of BMP9 predicted by NicheNet (Figure 6B). Endoglin knockdown reduced Eng expression compared with the negative control, and also decreased the expression of Cd5l, the glycoproteins secreted by CLEC4$F^{+}$ Kupffer cells [35]. siRNA_709 additionally downregulated Cd206 (encoded by Mrc1), which is associated with the macrophage repair phenotype [36]. siRNA_869 reduced Tgm2 expression, which is related to metabolic reprogramming and the anti-inflammatory effects of macrophages [37,38] (Figure 6C). These findings suggest that restoration of BMP signal-receiving capacity during the repair phase may be mechanistically linked to the functional reprogramming of hepatic macrophages during tissue recovery.

### 2.6. Human AILDs Macrophages Mirror Disease-Specific Maturation Patterns

**Figure 7:**
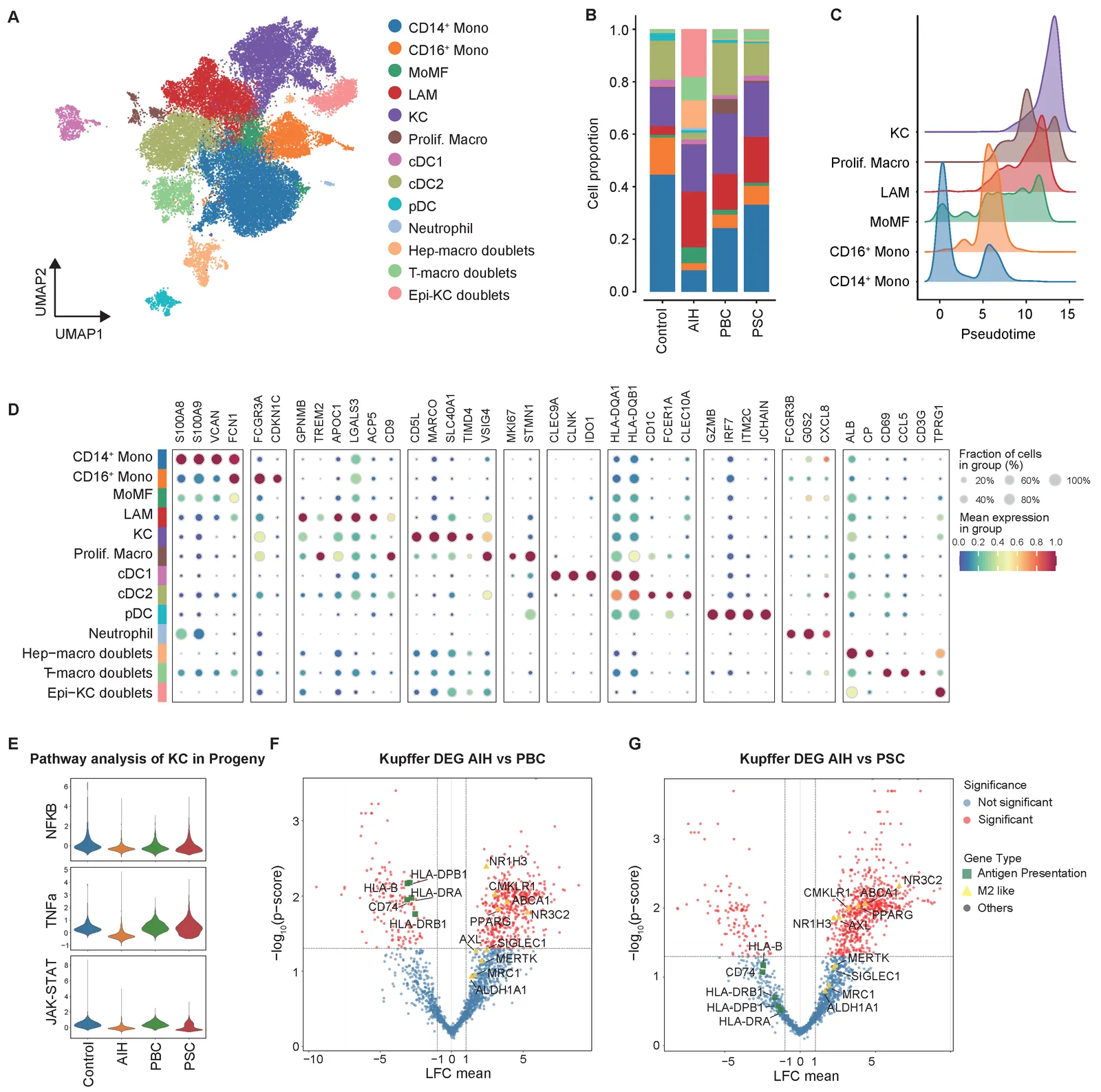
AILD patients’ macrophage phenotypes. (**A**) UMAP plot of myeloid cells. (**B**) Bar plots showing the cell proportion of liver myeloid cells. (**C**) Ridge plot showing Mono/Macro subtype distribution along pseudotime. (**D**) Bubble plot of myeloid cell markers. (**E**) Violin plot showing pathway enrichment analysis of KC, as inferred by Progeny. (**F**) Volcano plot showing DEGs of KC between AIH and PBC. (**G**) Volcano plot showing DEGs of KC between AIH and PSC. sigDEGs:FDR<0.05,$\;|\log_{2}\mathrm{FC}|>1$.

To examine whether the disease-associated shift in macrophage differentiation status observed in our mouse models can be recapitulated in AILDs patients, we curated myeloid cells from publicly available samples, including AIH (snRNA-seq and CITE-seq), PBC (scRNA-seq), PSC (scRNA-seq), and healthy controls (scRNA-seq). Following annotation, we corrected batch effects across experiments and integrated the datasets using scANVI, obtaining 31,925 cells with 3534 doublets arising predominantly from the snRNA-seq AIH batch and residing outside the main cluster (Figure 7A and Figure S6A,B). We then examined the proportions, differentiation trajectories, and defining markers of the macrophage subsets and found patterns that were similar to those seen in the mouse models (Figure 7B–D). The LAM subset was consistently expanded across all three disease subtypes. In the two cholangiocyte-targeting diseases (PBC and PSC), the liver was infiltrated by a greater proportion of monocytes, which were at an early stage of the differentiation trajectory, highly expressing the alarmins S100A8 and S100A9 (Supplementary Figure S6C). The cholestatic Kupffer cells exhibited higher inflammatory pathway scores (Figure 7E) and elevated expression of multiple HLA molecules and CD74, showing a phenotype more closely linked to antigen presentation and activation. In contrast, KCs from AIH patients upregulated a panel of efferocytosis-related molecules (MRC1, MERTK, AXL) and key nuclear factors (NR1H3, PPARG) (Figure 7F,G), a phenotype that closely mirrored the efferocytic, anti-inflammatory KC signature observed in our mouse models. Together, these findings demonstrate that the disease-specific macrophage phenotypes identified in mice are conserved across species and recapitulated in human AILD patients, particularly the correlation between intrahepatic cholestasis and immature monocyte accumulation.

## 3. Discussion

AILDs comprise hepatocyte-targeted AIH and cholangiocyte-targeted PBC and PSC, which differ in their response to immunosuppression therapy and clinical prognosis. However, no cellular atlas yet compares the three disease subtypes to figure out what molecular mechanisms cause the differences in therapy tendency and cirrhosis progression.

In this study, we integrated scRNA-seq data from AILDs mouse models to understand the immune heterogeneity determined by different target cells of injury. Cell–cell communication analysis indicated that T cells acted as the main signal-receiving compartment in the AAV8-IL12 hepatitis models but were less cytotoxic in the cholestatic models, while myeloid cells were the principal senders in all models. In AAV8-IL12 livers, PD-$1^{hi}$ CD8 EM retained GZMB and IFNγ production, suggesting that PD-1 here marks sustained activation rather than terminal exhaustion. In the dnTGFβRII ${\mathrm{Aire}}^{-/-}$ mouse model of AILDs, CAR-T-mediated depletion of the PD-$1^{+}$CD$8^{+}$ T cell subset achieved histological remission, indicating that they are actively pathogenic effectors [39]. Our data may clarify why the hepatitic niche, unlike the cholestatic niche, contains more targetable adaptive-immune nodes.

Macrophage subclustering revealed a shared loss of ResKC across models but context-dependent compensatory routes. In AIH models, monocytes progressed towards MoKC, with the proliferative subpopulation also serving as a supplement, whereas in cholestasis, they accumulated as Ly6$C^{hi}$ monocytes and ${\mathrm{Vcan}}^{+}$ cells, a pattern we confirmed by flow cytometry as an excess of Ly6$C^{hi}$${\mathrm{MHCII}}^{lo}$ cells. Trajectory analysis resolved this conversion into progenitor, inflammatory, resolution, and residency gene clusters, macrophages in the late stage of pseudotime acquire efferocytic (Mertk, Cd36, Stab1), antioxidant (Selenop, Hmox1), and reparative (Igf1) programs coinciding with LXR activation, which has been reported to restrain inflammatory gene expression and regulate cholesterol homeostasis and lipid synthesis toward alternative polarization [40]. Importantly, this divergence was conserved in human livers, where AIH Kupffer cells retained a homeostatic, efferocytosis-driven phenotype with NR1H3/PPARG activity, whereas PBC and PSC were characterized by S100A8/$9^{hi}$ monocyte accumulation and antigen-presenting, inflammatory Kupffer cells. LAM has been regarded as a conserved response to lipid- and debris-rich niches [41], and we observed their expansion in every AILD model and in human data, expanding the applicability of pathological cell subpopulations.

**Figure 8:**
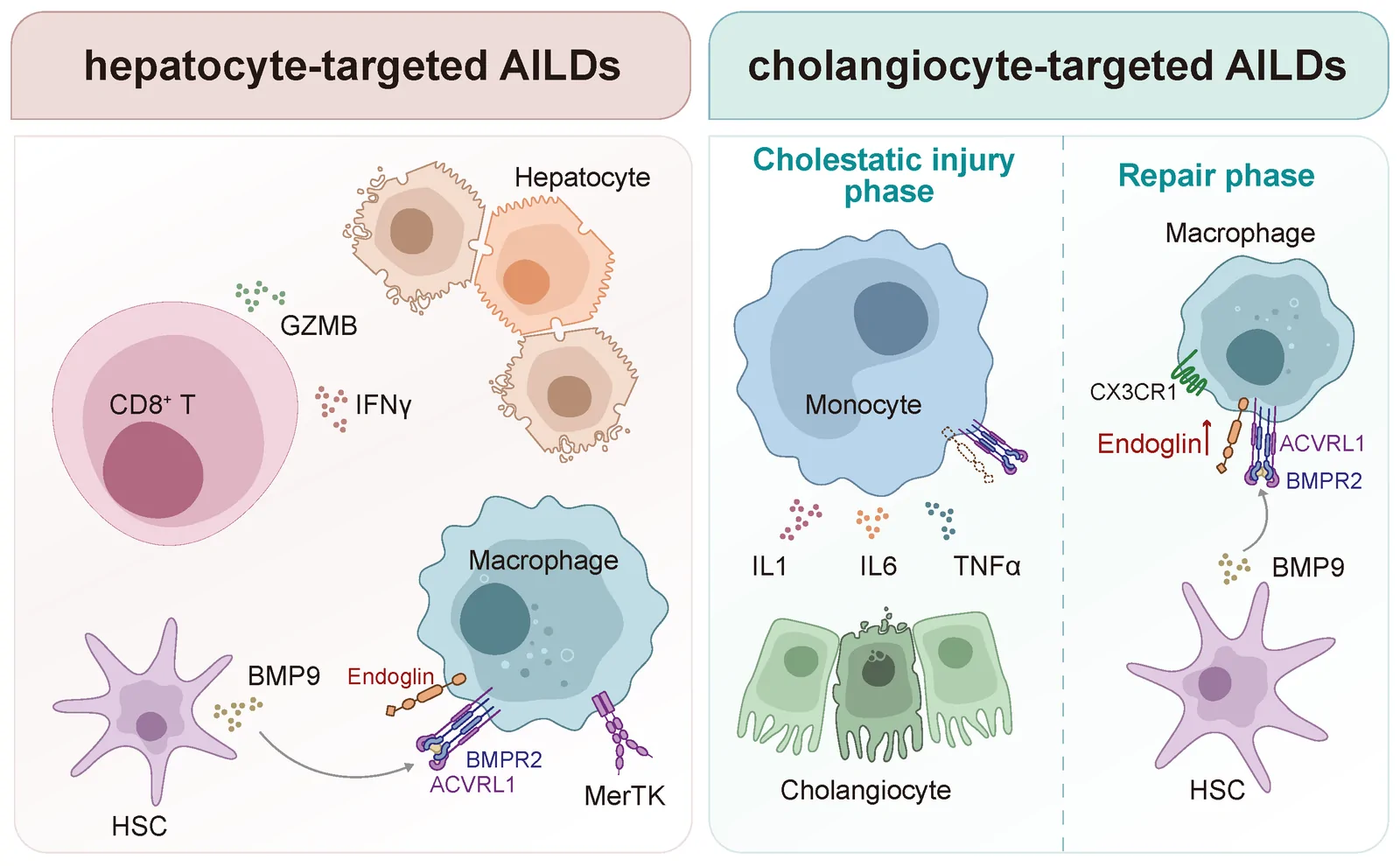
Distinct immune microenvironments in hepatocyte- and cholangiocyte-targeted autoimmune liver diseases. Hepatocyte injury is characterized by activated, non-exhausted CD$8^{+}$ T cells within a myeloid-derived network of activating and counter-regulatory signals. Cholangiocyte injury is dominated by macrophages arrested at an immature, pro-inflammatory monocyte stage with impaired Acvrl1/Eng signaling axis, preventing maturation toward KCs. DDC withdrawal reverses this block, indicating that macrophage maturation arrest is reversible upon niche improvement, suggesting that cholangiopathies may represent a disorder of arrested macrophage maturation, and endoglin serves as a potential therapeutic target.

Three independent analyses (NicheNet, CellChat, and CellOracle) identified a Bmp–Acvrl1/Eng–Fosb module. Fosb was the only candidate TF whose in silico deletion reproduced the Id3 phenotype, which is a Kupffer cell lineage-determining factor [28], marking it as a key link between niche signals and Kupffer identity. Prior KC-replenishment studies mainly used Clec4f-driven depletion models, where the intact niche lets monocyte-derived cells reliably become KCs within 1–2 weeks [13,29,31,42]. Unlike ablation models, cholestatic KC-replenishment deficit emerged from a disrupted niche, where HSC-derived Gdf2(Bmp9) signaling to Acvrl1/Eng receptors on macrophages was itself dysregulated. Especially impaired endoglin function compromises the efficient capture of BMP9 [33]. This distinction may account for the divergent outcome we observed, in that monocyte recruitment was abundant yet largely uncoupled from the acquisition of a mature KC program, suggesting that niche integrity and maturation blockade, rather than progenitor availability, constitute the rate-limiting step in KC repopulation under chronic biliary injury. Notably, during the recovery period in the cholestasis mouse model, Cx3cr$1^{+}$ macrophages, a subset transcriptionally resembling MoKCs, which promoted a restoration phenotype and immunomodulatory effect, expanded and partially replenished the depleted KC compartment. The reparative function and immunosuppressive effects of Cx3cr$1^{+}$ macrophages have been reported in recent studies [43,44], and our data emphasize their positive role during the repair phase of cholestatic liver injury. They also upregulated the expression of Eng, indicating that monocyte-to-KC differentiation was not intrinsically abrogated but had been held in abeyance and could resume once niche signaling was restored (Figure 8).

More broadly, BMP signaling in the liver has recently emerged as an area of active interest. Its functional outcome is highly context-dependent, a versatility that has attracted growing attention to BMP signaling as both a candidate therapeutic target and a source of circulating biomarkers for disease staging, but this attention has mainly focused on metabolic liver disease and fibrosis [45,46,47,48]. However, studies of endoglin as an auxiliary receptor have primarily focused on endothelial cells and fibroblasts, with disease-related research largely centered on circulatory system diseases and tumors. Although Eng expression has been reported in the monocyte/macrophage lineage and implicated in regulating macrophage polarization and phagocytic function [49,50,51], the relationship between Eng and hepatic diseases remains largely unexplored. To date, our study is the first to link the dysregulation of BMP signaling with the pathogenesis and heterogeneity of macrophages in AILDs, suggesting that the cholestatic environment impairs macrophage responsiveness to BMP signaling, leading to an imbalance in functional polarization, validated by down-regulating Eng expression and Acvrl1 downstreaming scores.

We are aware of several limitations in this study. First, although scVI/scANVI integration was applied, batch effects across datasets generated by different protocols may not be fully eliminated, and some inter-model differences could partly reflect technical differences. Second, as our AAV8-IL12 scRNA-seq dataset was not enriched for non-immune cell populations such as hepatocytes, the inferred non-immune-to-macrophage interactions were predicted primarily based on public reference datasets rather than matched validation; hepatocyte-enriched sequencing or functional assays are needed to confirm these signaling axes. Third, while the Bmp-acvrl1 signaling axis has been supported by prior literature, the downstream involvement of Eng and Fosb in mediating impaired macrophage maturation in our model remains inferred from transcriptomic and receptor-ligand analyses rather than functionally validated; whether Eng and Fosb are causally required for the arrested differentiation phenotype awaits direct experimental confirmation. Finally, human cohort data were cross-sectional, treatment-unmatched, and lacked in vitro validation; larger prospective, treatment-annotated cohorts are therefore needed to establish a definitive correlation between specific macrophage subpopulations and clinical treatment response, and to test whether macrophage maturation status predicts immunosuppressive therapy.

## 4. Materials and Methods

### 4.1. Animal Studies

Mdr$2^{-/-}$ mice were gifts from Dr. Jin Chai (Southwest Hospital, Third Military Medical University (Army Medical University)), and C57BL/6 mice were obtained from Shengchang Biotechnology Co., Ltd. (Shanghai, China). The mice were housed in individually ventilated cages under SPF conditions, with temperatures stabilized between 20– $26^{\circ }$C, humidity levels ranging from 40% to 70%, and on a 12 h light/dark cycle. Female mice aged 8–10 weeks received a 0.1% 3,5-diethoxycarbonyl-1,4-dihydrocollidine (DDC) diet for two weeks or were injected via the tail vein with AAV8-IL12, produced by Obio Technology (Shanghai, China), at a dose of 5 × $10^{10}$ viral genomes/kg to induce autoimmune hepatitis [52] and then sacrificed at four weeks. Specifically, the plasmid maps of the pAAV vectors were provided in Supplementary Figure S7A. The adenoviral empty-vector control group was generated and completed during our preliminary experiment; liver pathology data was shown in Supplementary Figure S7B and were indistinguishable from those of healthy mice. Livers were collected for histology, immunophenotyping, and single-cell RNA sequencing. All procedures involving animals received approval from the Institutional Animal Care and Use Committee of Shanghai Jiao Tong University School of Medicine.

### 4.2. Single-Cell Library Preparation and Sequencing

At week 4, AAV8-IL12-treated mice were euthanized by cervical dislocation, and liver tissues were harvested and maintained in cold DPBS (Thermo Fisher, Bellefonte, PA, USA, Cat# 14190144). Tissues were minced and enzymatically dissociated at $37^{\circ }$C with gentle shaking using 0.25% trypsin (Thermo Fisher, USA, Cat# 25200-072) and 10 μg/mL DNase I (Sigma, München, Germany, Cat# 11284932001). Cell suspensions were filtered through a 40 μm cell strainer, followed by red blood cell lysis (Thermo Fisher, USA, Cat# 00-4333-57) and washing in DPBS containing 0.4% FBS (Thermo Fisher, USA, Cat# SV30087.02). Liver immune cells were enriched by magnetic bead selection for CD$45^{+}$ cells (STEMCELL Technologies, Vancouver, BC, Canada, Cat# 18945) and then subjected to single-cell RNA sequencing on the DNBseq platform with the DNBelab C TaiM 4 V3.0 (BGI Genomics, Shenzhen, China), according to the manufacturer’s instructions.

### 4.3. Integration of scRNA-Seq Data with Public Databases

Our raw sequencing reads underwent processing with dnbc4tools (v2.1.2) to produce gene-barcode matrices aligned to the mouse reference genome (mm10). External publicly available disease models included concanavalin A (ConA)-induced acute AIH model (GSE287737 [3]), 2-octynoic acid coupled to bovine serum albumin(2OA–BSA) induced PBC (CRA017680 [8]), and DDC-induced PSC (GSE230085 [53]), profiled during the recovery phase at 2 weeks post-treatment, refer to the table for the integrated data structure (Table 1) Downstream analyses were performed using Scanpy (v1.11.4) in Python (v3.10.18). Doublets were identified via Solo, a deep learning-based doublet detection method. Since the dataset combined different experimental batches with varying sequencing depths, quality control was performed using a combination of adaptive median absolute deviation (MAD)-based filtering and predefined thresholds. Cells with outlying total_counts or n_genes_by_counts were excluded, together with cells showing high mitochondrial (10%) or hemoglobin (5%) gene proportions. After quality control, 73,982 high-quality CD$45^{+}$ cells remained for further analysis. From the six conditions mentioned above: Control (*n* = 6, number of cells = 16,617), AIH_ConA (*n* = 2, number of cells = 13,184), AIH_IL12 (*n* = 1, number of cells = 7236), PBC (*n* = 2, number of cells = 11,027), PSC (*n* = 2, number of cells = 12,334), PSC_Recovery (*n* = 3, number of cells = 13,584). The data were log-normalized, and 3000 highly variable genes were selected. Dimensionality reduction and batch integration were conducted using scVI (v1.3.3), with experimental batch included as a covariate.

To validate the phenotypes identified in mouse models within liver samples from patients with AILDs, the publicly available sequencing samples included 10 healthy controls, 7 PBC samples, 8 PSC samples, and mixed AIH samples, sourced from four databases (GSE136103 [54], HRA008003 [8], GSE247128 [7], the Research Data Repository of the University of Hamburg under DOI 10.25592/uhhfdm.18135 [55]), refer to the table for the integrated data structure (Table 2). The analysis workflow is similar to that used for mouse data. After quality control, we integrated a total of 31,925 myeloid cells with the following specific distribution: HC (*n* = 10, number of cells = 8447), PBC (*n* = 7, number of cells = 4560), PSC (*n* = 8, number of cells = 11,795), and AIH (*n* = 10 for CITE-seq and *n* = 6 for snRNA-seq, number of cells = 7123). To assess batch correction reliability across different sequencing technologies, we benchmarked integration methods using scib-metrics (v0.5.6), comparing unintegrated PCA, scVI, scANVI, Harmony, and Scanorama embeddings. scANVI achieved the best overall performance with the highest aggregate score, and its embeddings were subsequently used to generate UMAP visualizations.

### 4.4. Cell Annotation Analysis

To cluster cells, we utilized the Leiden algorithm. For visualization purposes, we applied Uniform Manifold Approximation and Projection (UMAP). The annotation of immune cell subgroups was conducted based on differentially expressed genes (DEGs) identified via the sc.tl.rank_genes_groups function, along with consensus markers for accurate annotation.

### 4.5. Cell–Cell Communication Analysis

The integrated mouse AILDs scRNA-seq data were analyzed using the CellChat R package (v2.2.0) to explore cell–cell communication. Communication probabilities were calculated, and low-frequency events were filtered out. The netAnalysis_signalingRole_scatter function was then used to define the roles of immune cell subsets within the communication network, such as whether they serve as signal senders or receivers. Visualization of cell–cell communication strength, mediated by various ligand–receptor pairs, was achieved through netVisual_bubble and netVisual_chord_gene. Additionally, the NicheNet R package (v2.2.1.1) was employed to identify potential microenvironment-derived signals involved in macrophage maturation regulation. NicheNet predicts ligands from sender cells that can influence target gene expression in receiver cells. DEGs between early- and late-stage macrophages along the pseudotime trajectory were identified as the gene set of interest. A ligand–target heatmap was then generated to highlight candidate ligands that may drive macrophage differentiation toward the late stage, along with their predicted intrinsic target genes in macrophages.

### 4.6. Trajectory Analysis

Monocle R package (v2.24.0) was used to assess the differentiation trajectory and developmental potential of Ly6$C^{hi}$ classical monocytes toward macrophages. Following detailed subclustering of the monocyte/macrophage populations, expression matrices of Ly6$C^{hi}$ monocytes and their putative differentiated subpopulations were extracted and analyzed using Monocle2. Dimensionality reduction was performed with the DDRTree algorithm. Cells were ordered along the inferred trajectory using the orderCells function, which assigned pseudotime values and defined distinct differentiation states. The plot_cell_trajectory function was used to visualize pseudotime trajectories across cell subpopulations, whereas plot_complex_cell_trajectory was used to visualize the branching tree structures of cell differentiation trajectories across different disease models.

To enhance statistical power and enrich data presentation formats, we separated macrophage cells into early and late stages using a pseudotime cutoff of 10, which represents the median of the pseudotime distribution. Immature monocytes exhibited pseudotime values below 10, while mature phenotype showed pseudotime values above 10; therefore, 10 was designated as the threshold. Each sample was artificially divided into two pseudo-samples representing early and late stages, which were then subjected to paired comparisons and visualized through volcano plots.

### 4.7. Enrichment Analysis

Key analysis of macrophage subpopulations’ functions was performed using the Scanpy native function sc.tl.score_genes to evaluate macrophage subgroups based on gene sets from the established literature, helping to define their characteristics. Additionally, Decoupler (v2.1.4) is used to aggregate gene expression counts across all cells of the same type within a sample, forming pseudo-bulk, with low-quality pseudo-bulk samples (min_cells < 10, min_counts < 1000) filtered out. Statistical comparisons are performed with the PyDESeq2 package(v0.5.2), where each cell type is treated as the ’target’, and the sum of all other cell types within the sample as the ’background’. A paired design identifies genes significantly upregulated in specific macrophage subpopulations. A prerank list of DEGs is created based on adjusted *p*-values and log2FoldChange, followed by enrichment analysis using the gp.prerank function in GSEApy (v1.1.11).

### 4.8. Regulatory Network Inference and Virtual Knockout

To forecast the regulatory network controlling macrophage maturation phenotypes, we used CellOracle (v0.20.0) for gene regulatory network inference and in silico transcription factor perturbation. We simulated the deletion of the candidate transcription factor and created vector field plots to visualize the predicted directions and cell-state transitions in Ly6$C^{hi}$ monocytes and their potential differentiated subpopulations.

### 4.9. Flow Cytometry

Mouse liver mononuclear cells were isolated through mechanical dissociation followed by Percoll gradient centrifugation. The liver was dissociated using the gentleMACS Dissociator (Miltenyi Biotec, Bergisch Gladbach, Germany), and fibrous tissue was filtered through a 70 μm strainer. For centrifugation, cell suspensions from liver digestion were layered in 33% Percoll (Cytiva, Marlborough, MA, USA, Cat# 17089101) and spun at 900× *g* for 30 min without brake, resulting in a density-based separation with mononuclear cells enriched in the bottom pellet. To assess T effector function via intracellular cytokine staining, cells were first surface-stained with anti-mouse CD3 (BD Bioscience, San Diego, CA, USA, Cat#564378), NK1.1 (Biolegend, San Diego, CA, USA, Cat#108749), CD8 (BD Bioscience, USA, Cat# 553031), PD-1 (BD Bioscience, USA, Cat# 566514), and live/dead dye (BD Bioscience, USA, Cat# 565388) at $4^{\circ }$C for 30 min in the dark, then fixed and permeabilized before staining with GZMB (Invitrogen, Carlsbad, CA, USA, Cat#12-8898-82) and IFNγ (BD Bioscience, USA, Cat#562303). For macrophage phenotype analysis, cells were surface-stained with anti-mouse CD11b (BD Bioscience, USA, Cat# 740861), CD64 (BD Bioscience, USA, Cat# 558455), F4/80 (eBioscience, San Diego, CA, USA, Cat#56-4801-82), MHCII (BD Bioscience, USA, Cat#107639), Ly6C (BD Bioscience, USA, Cat#561085), CLEC2 (Biolegend, USA, Cat#146105), VSIG4 (eBioscience, USA, Cat#25-5752-82), and live/dead dye (BD Bioscience, USA, Cat# 565388). Flow cytometric acquisition was performed using the spectral flow cytometer (Cytek Biosciences, Fremont, CA, USA), and data were analyzed with FlowJo software (v10.9.0). In addition, the flow-cytometry gating strategy is shown in Supplementary Figure S7C,D.

### 4.10. Bone Marrow-Derived Macrophages (BMDM) Culture and KC-like Phenomenon Induction

We used a DLL4 + BMP9 culture system to induce a KC-like phenotype in monocytes in vitro [29,31]. Femurs and tibias from 7-week-old male C57BL/6J mice were flushed with DMEM (Gibco, Waltham, MA, USA, Cat# 11995065). Bone marrow cells were seeded in 10 cm non-tissue culture plates in DMEM supplemented with 10% FBS (Gibco, USA, Cat# A5256701), 30 ng/mL M-CSF (PeproTech, Cranbury, NJ, USA, Cat# 315-02-10UG), 100 U/mL penicillin/streptomycin (Gibco, USA, Cat# 15140122), and L-glutamine (Gibco, USA, Cat# 35050061). After 3 days of differentiation, non-adherent cells were washed off with room-temperature DMEM, and adherent bone marrow progenitor cells were detached with EDTA (Invitrogen, USA, Cat# 15575020) and reseeded onto tissue culture-treated plates coated with 1 μg/mL DLL4 (MCE, Princeton, NJ, USA, Cat# HY-P75292). Cells were then stimulated with 50 ng/mL recombinant BMP9 (PeproTech, Cranbury, NJ, USA, Cat# 120-07-2UG), added 48 h after transfection with siRNA targeting Eng. Cells were collected for RT-qPCR 24 h after BMP9 stimulation.

### 4.11. Transfection with siRNA Targeting the Endoglin Gene

Adjust the bone marrow cell density to $8\times 10^{5}$ before transfection, and seed the cells into a 12-well plate such that the cells are 70% confluent at the time of transfection. Perform the transfection according to the transfection reagent manufacturer’s instructions (Yeasen, Shanghai, China, Cat# 40807ES), roughly as follows: dilute the siRNA to 20 μM, then pipette 2.5 μL of the siRNA solution and 12.5 μL of the Enhancer solution, and mix thoroughly by pipetting. Add 5 μL of RNAiBoost for in vitro transfection and mix thoroughly by pipetting. Incubate at room temperature for 5 min. After incubation, add 30 μL of opti-MEM to the mixture and mix thoroughly. Then add the resulting 50 μL transfection complex to one well of the 12-well plate, and gently rock the culture plate to mix the liquid. Set up two siRNA experimental groups and one negative control group, with three replicate wells per group. Two pairs of siRNA sequences targeting mouse Eng are shown in Table 3.

### 4.12. Immunofluorescence Staining

Paraffin-embedded mouse liver sections were subjected to immunofluorescence staining to assess the in situ distribution and phenotypic characteristics of macrophages. Anti-mouse MerTK (Abcam, Cambridge, UK, Cat# ab300136) and F4/80 (Abcam, Cambridge, UK, Cat# ab300421) antibodies were used for staining with tyramide signal amplification (TSA), according to the manufacturer’s instructions (Absin, Shanghai, China, Cat# abs50012-2OT). Nuclei were counterstained with DAPI (SouthernBiotech, Birmingham, AL, USA, Cat# 0100-20) prior to imaging.

### 4.13. Total RNA Extraction and RT-qPCR

Mouse liver mononuclear cells were isolated as described above. To enrich macrophage populations, cells were first incubated with a biotin-conjugated anti-mouse F4/80 antibody (Biolegend, USA, Cat# 123106), then with anti-biotin microbeads (Miltenyi Biotec, Bergisch Gladbach, Germany, Cat# 130-090-485). F4/80-positive cells were isolated via magnetic-activated cell sorting (MACS) according to the manufacturer’s instructions. Purity and activity were verified by flow cytometry. The enriched macrophage pellet was then used to extract total RNA using the TRIzol (TaKaRa, Shiga, Japan, Cat# 9109) method. cDNA reverse transcription synthesis (TaKaRa, Japan, Cat# RR036A) and qPCR (Vazyme, Nanjing, China, Cat# Q411-02) were performed according to the manufacturer’s protocol. The RT-qPCR primers are listed in Table 4. Gene expression was normalized to Actb and analyzed using the $2^{-\Delta \Delta C_{t}}$ method.

### 4.14. Statistical Analysis

Statistical analyses were conducted with GraphPad Prism (v10.4.2), R, and Python. We processed and analyzed scRNA-seq using R- and Python-based bioinformatics workflows, as previously described. Data from flow cytometry, multiplex immunofluorescence cell counting, and RT–qPCR are shown as mean ± standard error (SEM). Comparisons across three groups were made using one-way ANOVA, followed by Tukey’s test for multiple comparisons, assuming normal distribution and equal variances. Significance levels were set at * p<0.05, ** p<0.01, *** p<0.001, and **** p<0.0001.

## 5. Conclusions

In summary, by comparing hepatocyte- and cholangiocyte-targeted autoimmune liver injury within a single analytical framework, we show that different disease subtypes have distinct immune microenvironments. Hepatocyte injury was characterized by activated, non-exhausted CD$8^{+}$ T cells within a myeloid-derived network of activating and counter-regulatory signals. Cholangiocyte injury, in contrast, was dominated by macrophages arrested at an immature, pro-inflammatory monocyte stage, with failure to rebuild the Kupffer cell niche and reduced efferocytic and reparative gene programs. Multiple independent inference methods identified the Bmp–Acvrl1/Eng–Fosb module as a potential regulator of this maturation checkpoint. In the repair Phase of the Cholestasis model, reversal of the block suggested that impaired macrophage sensitivity to Bmp signaling is reversible after the niche improves. The same mono/macrophage polarity was conserved in human AIH, PBC, and PSC. Lastly, our findings reframe cholangiopathies as a potential disorder of arrested macrophage maturation and provide a rationale for microenvironment-tailored, rather than uniformly immunosuppressive, therapeutic strategies in AILDs, especially to restore sensitivity of Eng-mediated Bmp9 signaling.

## Figures and Tables

**Table 1:** Sources and relevant information on scRNA-seq data from liver tissues of AILD mouse models and controls.

Database Number	Sample Composition	Platform
AAV8-IL12 models *	1 chronic AIH model	MGI DNBseq
GSE287737	1 Control and 2 ConA-induced acute AIH model	Illumina NovaSeq 6000
CRA017680	2 Control and 2 2OA-BSA-induced PBC model	Illumina NovaSeq 6000
GSE230085	3 Control, 2 14-days-DDC-induced PSC model and 3 14-days-post-DDC-induced recovery model	Illumina NovaSeq 6000

* The AAV8-IL12 scRNA-seq data was performed in house and enrichment of CD$45^{+}$ cells.

**Table 2:** Sources and relevant information on sequencing data from liver tissues of AILD patients and controls.

Database Number	Sample Composition	Platform	Library Preparation Protocol
GSE136103	5 Healthy controls	Illumina HiSeq 4000	Single Cell 3′ Reagent Kits
HRA008003	5 Healthy controls and 5 PBC	Illumina NovaSeq 6000	Single Cell 3′ Reagent Kits
GSE247128	2 PBC and 8 PSC	Illumina HiSeq 2500	Single Cell 5′ Reagent Kits
AIH samples *	mixed AIH samples	Illumina NovaSeq 6000	CITE-seq and Single nuclei 3′ Reagent Kits

* The Research Data Repository of the University of Hamburg under DOI 10.25592/uhhfdm.18135.

**Table 3:** Sequences of siRNA duplexes against murine endoglin.

Against Murine Endoglin	Sense Strand ($5^{\prime }\to 3^{\prime }$)	Antisense Strand ($5^{\prime }\to 3^{\prime }$)
m_siRNA 709	CCUGGUUCAUCGACAUCAA	UUGAUGUCGAUGAACCAGG
m_siRNA 869	CCUGCUGUUUGUCAUCUAUAGCUUU	AAAGCUAUAGAUGACAAACAGCAGG

**Table 4:** RT-qPCR primer sequences used in the study.

Gene Name	Forward Primer ($5^{\prime }\to 3^{\prime }$)	Reverse Primer ($5^{\prime }\to 3^{\prime }$)
*Eng*	CAGCCAAAGTGTGGCAATCAGG	GCTACTCAGGACAAGATGGTCG
*Acvrl1*	CTTCTTGGTGCAGAGGACGGTA	CGTGAGGAGAAAATCTTGACCGC
*Bmpr2*	AGAGACCCAAGTTCCCAGAAGC	TCTCCTCAGCACACTGTGCAGT
*Id3*	CACTTACCCTGAACTCAACGCC	CCCATTCTCGGAAAAGCCAG
*Tgm2*	GAAGGAACACGGCTGTCAGCAA	GATGAGCAGGTTGCTGTTCTGG
*Cd5l*	GAGGACACATGGATGGAATGT	ACCCTTGTGTAGCACCTCCA
*Mrc1*	GTTCACCTGGAGTGATGGTTCTC	AGGACATGCCAGGGTCACCTTT
*Actb*	CATTGCTGACAGGATGCAGAAGG	TGCTGGAAGGTGGACAGTGAGG

## Data Availability

The single-cell RNA-sequencing data generated from the AAV8-IL12 mouse model are available from the corresponding author upon reasonable request. Publicly available datasets analyzed in this study are available in the GEO repository (https://www.ncbi.nlm.nih.gov/geo/, accessed on 1 January 2026); the GSA and GSA-Human repositories at the National Genomics Data Center, China National Center for Bioinformation (https://ngdc.cncb.ac.cn/gsa/; https://ngdc.cncb.ac.cn/gsa-human/, accessed on 1 January 2026), and the Research Data Repository of the University of Hamburg (https://www.fdr.uni-hamburg.de/, accessed on 30 July 2026). The reference numbers can be obtained in Table 1 and Table 2.

## Supplementary Materials

The following supporting information can be downloaded at https://www.mdpi.com/article/10.3390/ijms27209323/s1.

## Abbreviations

The following abbreviations are used in this manuscript:

AILD	Autoimmune liver diseases
PBC	Primary biliary cholangitis
PSC	Primary sclerosing cholangitis
AIH	Autoimmune hepatitis
BMP	Bone morphogenetic protein
KC	Kupffer cell
JAK-STAT	Janus kinase–signal transducer and activator of transcription
ROS	Reactive oxygen species
LAM	Lipid-associated macrophage
AAV8	Adeno-associated virus serotype 8
MDR2	Multidrug resistance protein 2
DDC	3,5-diethoxycarbonyl-1,4-dihydrocollidine
ConA	Concanavalin A
2OA-BSA	2-octynoic acid coupled to bovine serum albumin
MoMF	Monocyte-derived macrophage
MoKC	Monocyte-derived Kupffer-like cell
LSEC	Liver sinusoidal endothelial cell
HSC	Hepatic stellate cell
RT-qPCR	Reverse transcription-quantitative polymerase chain reaction
scRNA-seq	Single-cell RNA sequencing
snRNA-seq	Single-nucleus RNA sequencing
CITE-seq	Cellular indexing of transcriptomes and epitopes by sequencing
