# Supplementary material for: Single-Cell Transcriptomic Analysis Reveals Distinct Pathogenic Subtypes in Hepatocyte- and Cholangiocyte-Directed Autoimmune Liver Injury
Source: Int J Mol Sci. 2026 Oct 20;27(20):9323. doi: 10.3390/ijms27209323 (PMC13705488; doi:10.3390/ijms27209323)
Supplement: Supplementary file 1 [file ijms-27-09323-s001.zip › ijms-4536911-supplementary.pdf]

Supplementary Figures

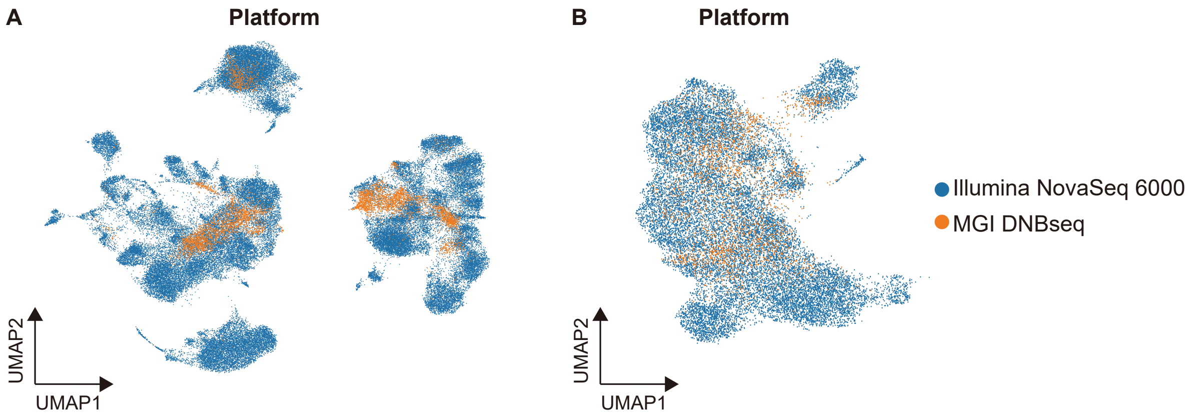

Supplementary Figure S1: UMAP plots of the sequencing platform indicate effective batch effect removal.(**A**) UMAP plot of CD45$^+$ cells. (**B**)UMAP plot of macrophage subtypes.

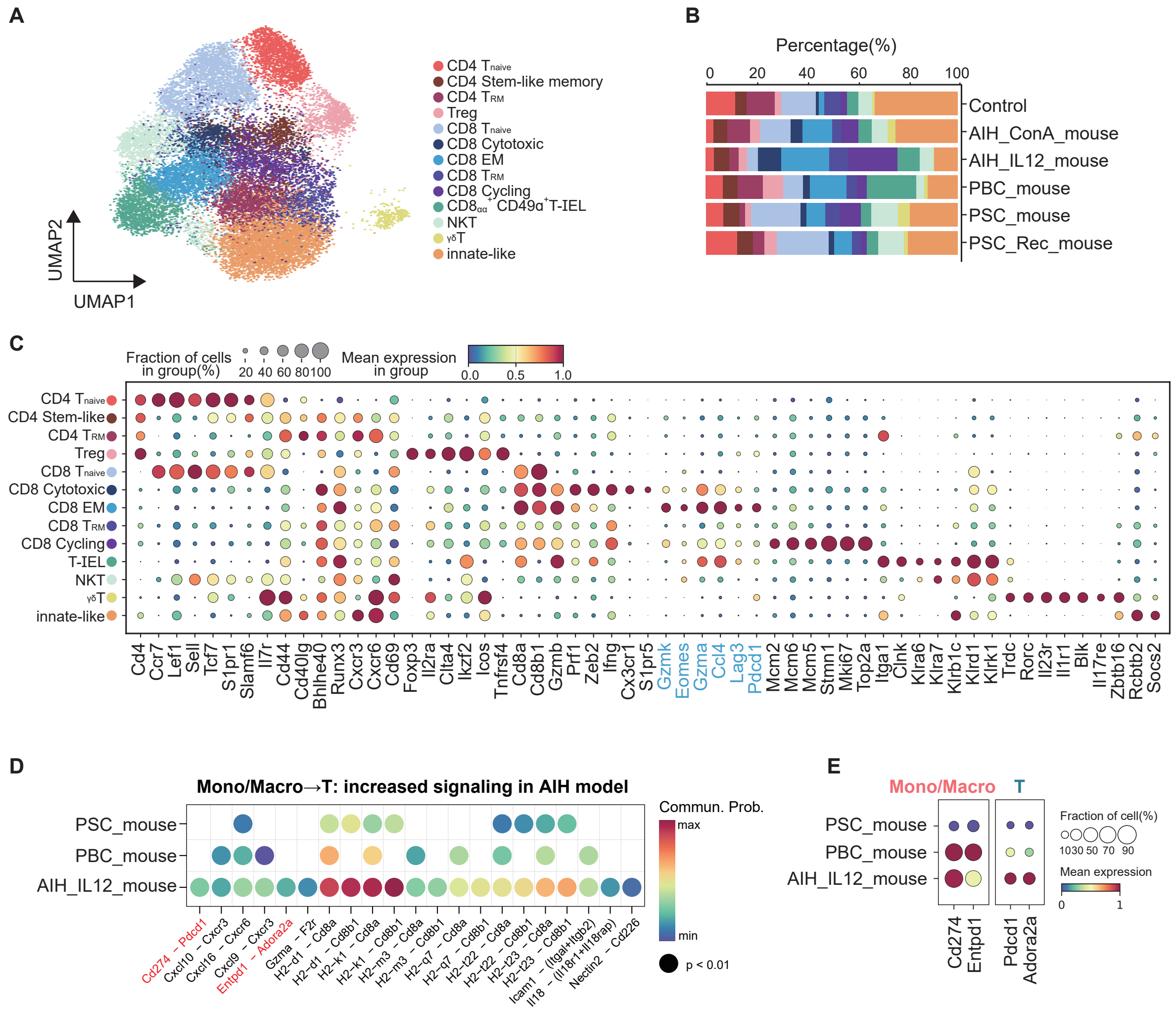

Supplementary Figure S2: T cell heterogeneity across different AILDs models.(**A**) UMAP plot of T cell clusters. (**B**)Bar plots showing the cell proportion of liver T cell subsets. (**C**) Bubble plot of T subcluster markers. (**D**) Bubble plot of ligand-receptor pairs. (**E**) Bubble plots showing gene relative expression.

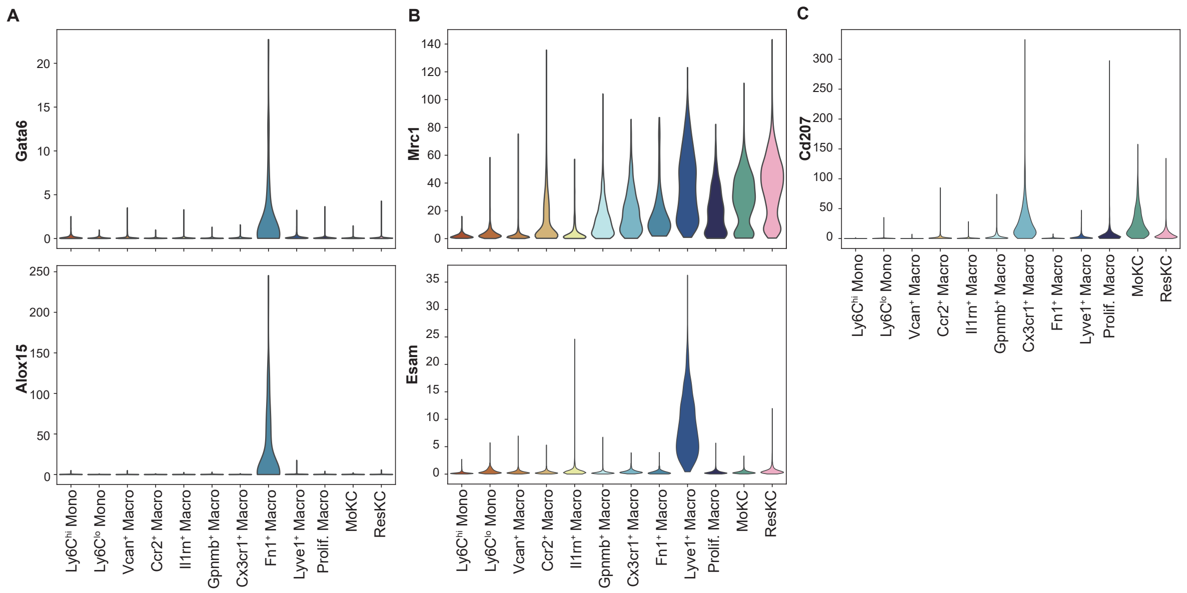

Supplementary Figure S3: Identify markers of macrophage subtypes. (**A**) Fn1$^+$ Macro with high expression of Gata6 and Alox15. (**B**) Lyve1$^+$ Macro with high expression of Mrc1 and Esam. (**C**) Cx3cr1$^+$ macrophages and MoKC both highly express Cd207.

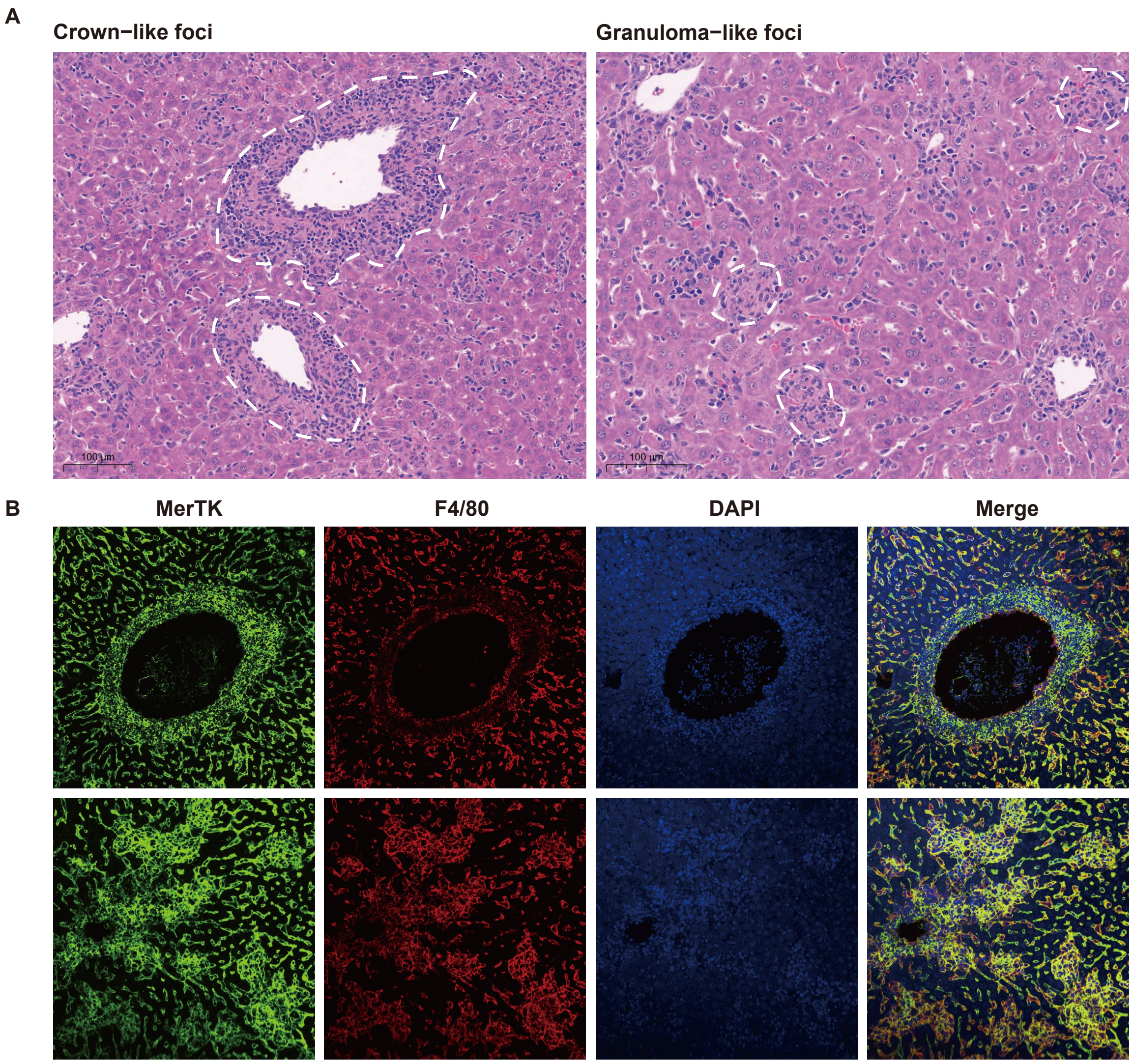

Supplementary Figure S4: Crown-like and granuloma-like foci in the liver of AAV8-IL12 models. (**A**) Representative images of H&E staining. (**B**) Representative images of immunofluorescence staining.

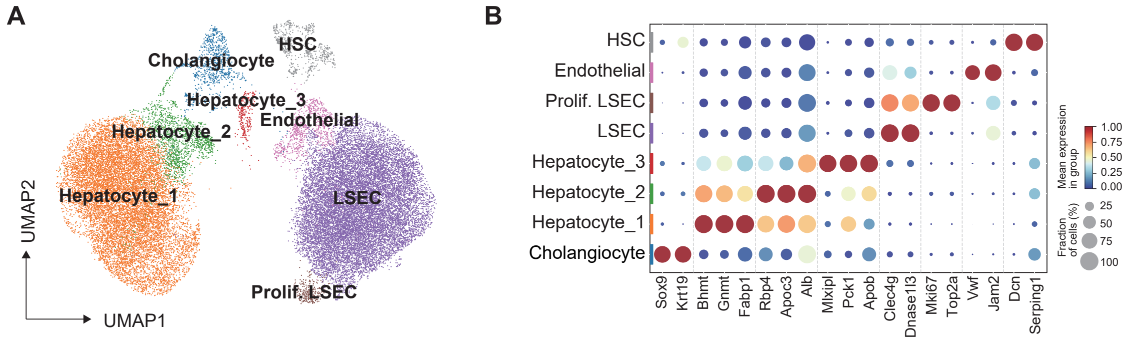

Supplementary Figure S5: The single-cell atlas of liver non-immune cells. (**A**)UMAP plot of non-immune cells. (**B**) Bubble plot showing markers of non-immune cells.

A

	Bio conservation					Batch correction					Aggregate score		
**Method**	Isolated labels	KMeans NMI	KMeans ARI	Silhouette label	cLISI	BRAS	iLISI	KBET	Graph connectivity	PCR comparison	Batch correction	Bio conservation	Total
**scANVI**	0.75	0.62	0.53	0.59	1.00	0.85	0.11	0.12	0.97	0.76	0.56	0.70	0.64
**scVI**	0.73	0.52	0.47	0.55	0.99	0.86	0.13	0.12	0.96	0.86	0.58	0.65	0.63
**X_pca_harmony**	0.60	0.13	0.07	0.47	0.92	0.69	0.10	0.10	0.66	0.63	0.44	0.44	0.44
**Unintegrated**	0.60	0.14	0.09	0.47	0.94	0.60	0.00	0.02	0.68	0.00	0.26	0.45	0.37
**X_scanorama**	0.49	0.14	0.08	0.47	0.88	0.57	0.03	0.01	0.44	0.00	0.21	0.41	0.33

B

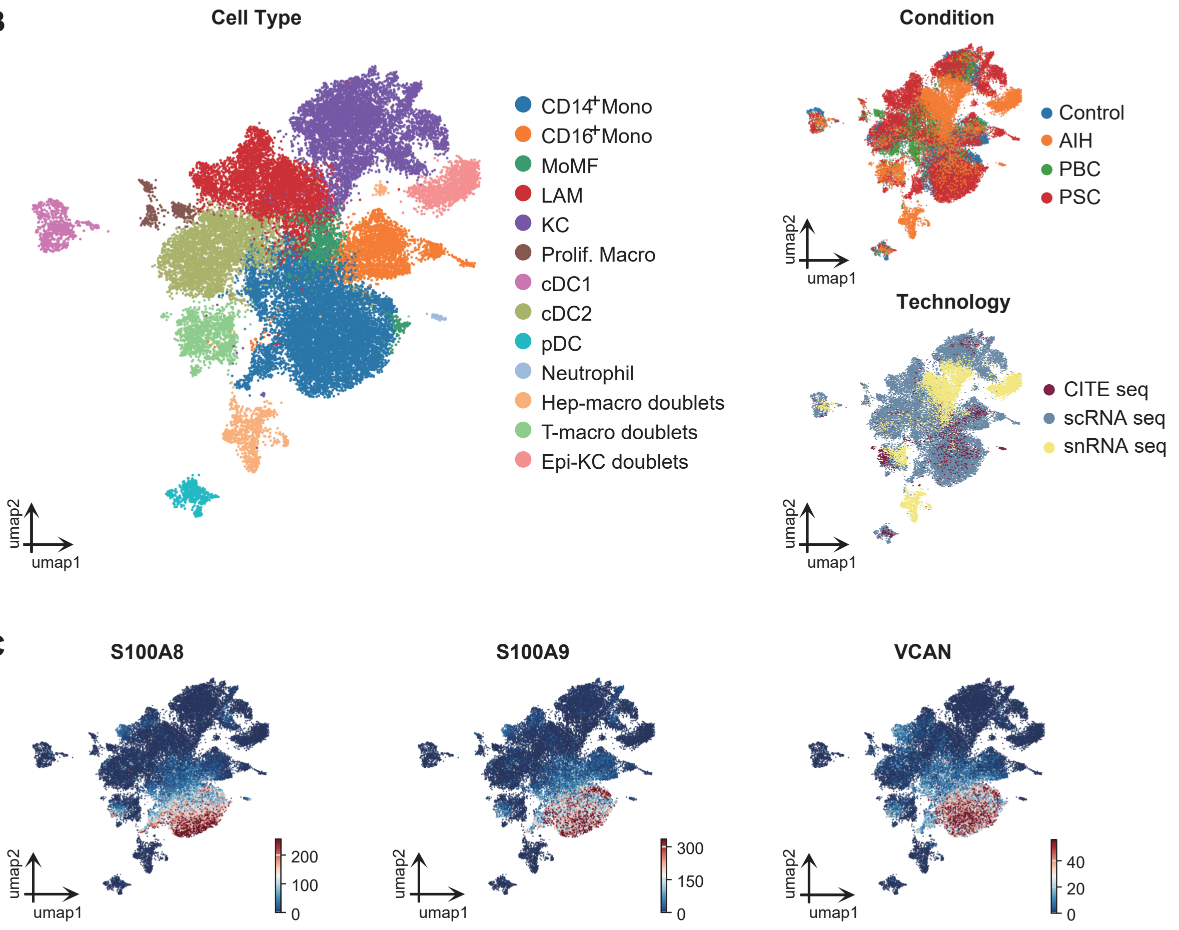

Supplementary Figure S6: Benchmarking myeloid cell integration in AILD patients using scib-metrics (**A**)Batch correction scores of different embeddings. (**B**) UMAP plots showing minor batch effects from non-biological factors on monocyte populations. (**C**) UMAP plots of markers in classical monocyte subsets.

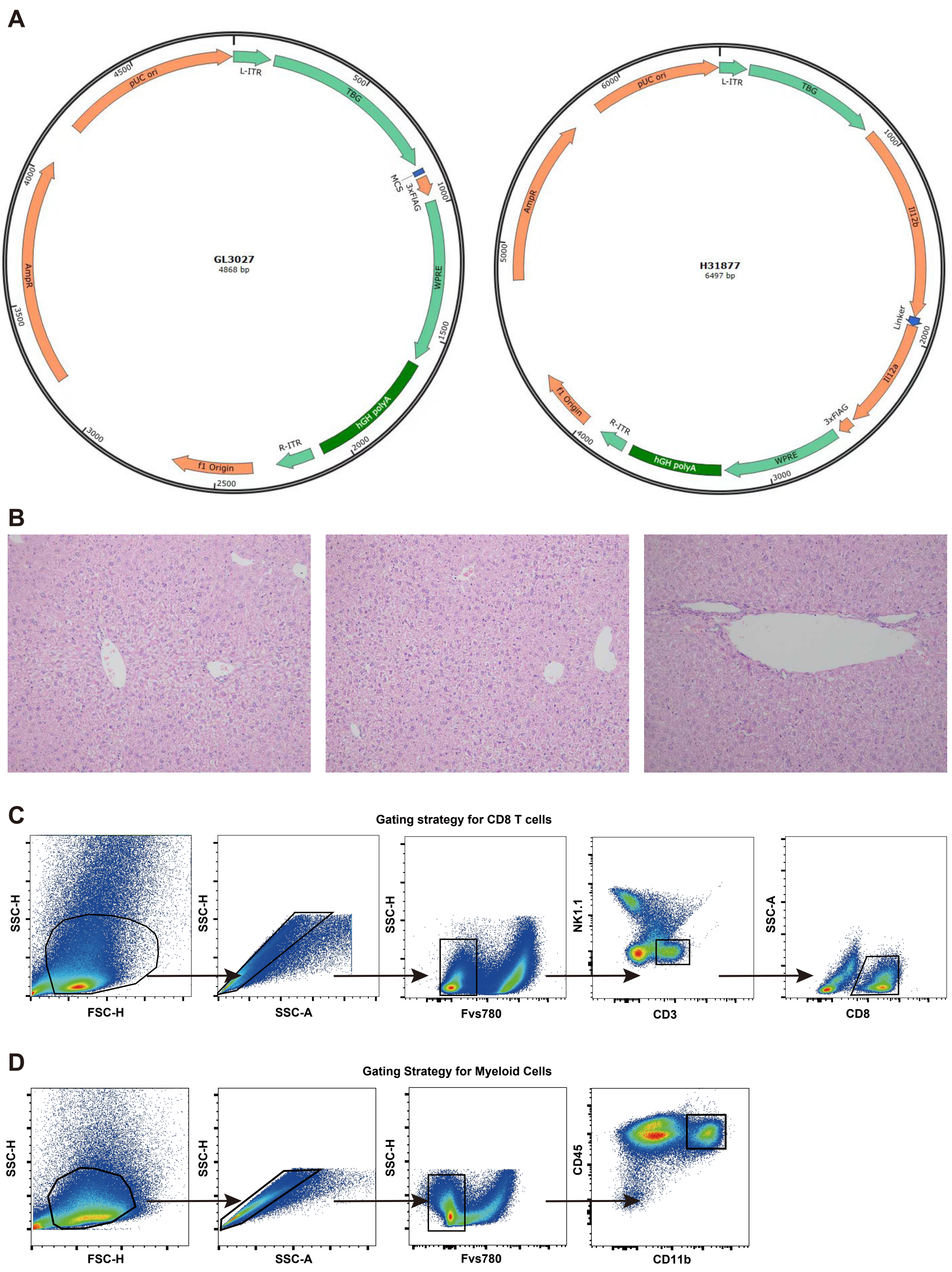

Supplementary Figure S7: Methodological supplement for in vivo animal experiments. (**A**)The plasmid maps. Both constructs utilized the hepatocyte-tropic AAV backbone with a tissue-specific promoter TBG to drive liver-directed transgene expression. The IL-12 construct (pAAV-TBG-IL12b-Linker-IL12a-3xFLAG-WPRE, right) encoded both IL-12 subunits linked by a flexible peptide, with a 3×FLAG tag for detection and WPRE for enhanced expression. The control vector (pAAV-TBG-MCS-3xFLAG, left) contained an empty MCS. Titers: control 2.25×10^{13} v.g./mL and IL-12 vector 1.80×10^{13} v.g./mL. (**B**) H&E histopathology of mice livers following control vector model construction. n=3. (**C**)Flow cytometry gating strategy of $CD8^+$ T cells. (**D**) Flow cytometry gating strategy of myloid cells.
